# Ascorbic acid tethered polymeric nanoparticles enable efficient brain delivery of galantamine: An *in vitro*-*in vivo* study

**DOI:** 10.1038/s41598-017-11611-4

**Published:** 2017-09-11

**Authors:** Kavita R. Gajbhiye, Virendra Gajbhiye, Imtiaz A. Siddiqui, Srikanth Pilla, Vandana Soni

**Affiliations:** 1 Department of Pharmaceutical Sciences, School of Engineering and Technology, Dr. Hari Singh Gour University, Sagar, MP 470003 India; 2 Department of Dermatology, University of Wisconsin, Madison, WI 53706 USA; 3 Automotive Engineering, Clemson University, Greenville, SC 29607 USA; 4 Materials Science & Engineering, Clemson University, Clemson, SC 29634 USA; 5 Present Address: Nanobioscience, Agharkar Research Institute, Pune, 411004 India

## Abstract

The aim of this work was to enhance the transportation of the galantamine to the brain via ascorbic acid grafted PLGA-*b*-PEG nanoparticles (NPs) using SVCT2 transporters of choroid plexus. PLGA-*b*-PEG copolymer was synthesized and characterized by ^1^H NMR, gel permeation chromatography, and differential scanning calorimetry. PLGA-*b*-PEG-NH_2_ and PLGA-*b*-mPEG NPs were prepared by nanoprecipitation method. PLGA-*b*-PEG NPs with desirable size, polydispersity, and drug loading were used for the conjugation with ascorbic acid (PLGA-*b*-PEG-Asc) to facilitate SVCT2 mediated transportation of the same into the brain. The surface functionalization of NPs with ascorbic acid significantly increased cellular uptake of NPs in SVCT2 expressing NIH/3T3 cells as compared to plain PLGA and PLGA-*b*-mPEG NPs. *In vivo* pharmacodynamic efficacy was evaluated using Morris Water Maze Test, Radial Arm Maze Test and AChE activity in scopolamine induced amnetic rats. *In vivo* pharmacodynamic studies demonstrated significantly higher therapeutic and sustained action by drug loaded PLGA-*b*-PEG-Asc NPs than free drugs and drug loaded plain PLGA as well as PLGA-*b*-mPEG NPs. Additionally, PLGA-*b*-PEG-Asc NPs resulted in significantly higher biodistribution of the drug to the brain than other formulations. Hence, the results suggested that targeting of bioactives to the brain by ascorbic acid grafted PLGA-*b*-PEG NPs is a promising approach.

## Introduction

Alzheimer’s disease (AD) is a deadly neurodegenerative disorder characterized by a progressive and permanent degeneration in different regions of the brain, particularly in the area which is important for memory and cognition. The neuropathological features of AD are characterized by enhanced production as well as accumulation of amyloid-β peptide (Aβ) in senile plaques which contribute to progressive neuro-degeneration. Therefore, AD leads to memory and cognitive impairment, language difficulties, anxiety, confusion, and mood swings. In the advanced stage of AD, patients are unable to execute motor functions and need the support of others to carry out essential activities.

Even though the correct pathogenesis of neuronal deterioration and cognitive impairment in AD is still ambiguous, the decline in central cholinergic neurotransmission is one of the most vital pharmacological and neurochemical facts related to AD. The literature clearly suggests that there is marked decrease in the activity of choline acetyltransferase (ChAT) in the AD brain, which is obligatory for the synthesis of acetylcholine (ACh)^1^. The degree of depletion of ChAT can be correlated with the severity of cognitive impairment. Cholinesterase inhibitors (ChEI) e.g. donepezil, rivastigmine, galantamine etc. have shown to avert hydrolysis of ACh and elevate its concentration in the synaptic cleft, which leads to increase in cholinergic transmission in AD brain. Galantamine is a plant alkaloid with potent acetylcholinesterase (AChE) inhibitor activity. Additionally it increases the sensitivity of nicotinic receptors for acetylcholine neurotransmitter. Long-term treatment of AD brain with galantamine retards cognitive disturbance as well as diminishes behavioral symptoms, mainly in patients with modest or advanced stages of the disease. Even if, the galantamine itself has the ability to cross the blood-brain barrier (BBB) from conventional formulations and reach the brain, a higher dose is essential to attain therapeutic levels due to its non-specificity for the brain. This non-specificity potentiate the peripheral drug burden, and galantamine is known to exert adverse cholinergic effects on peripheral organs like other cholinesterase inhibitors due to non-specific interaction^2, 3^. Therefore, targeted drug delivery strategies using nanotechnology is essential to augment brain specificity of galantamine and diminish the peripheral burden without compromising the patient compliance. The therapeutic dose required could easily be loaded in targeted nanoparticles and would not require the administration of a large amount of potentially toxic dose. A large number of NPs with different targeting ligands have been developed and employed for targeted brain delivery of bioactives^4^.

Ascorbic acid (ACS) plays an essential role in CNS as a cofactor in several enzymatic reactions related to the neurotransmitters processing and also as an antioxidant preventing neuro-degeneration. Sodium dependent-vitamin C transporters (SVCT2), expressed by neuroepithelial cells of the choroid plexus are involved in the transport of the reduced form of ascorbic acid. It has been established that ascorbic acid concentration attains up to 200-fold higher in some neurons, as compared with concentration in the bloodstream^5^. There are explicit physiological mechanisms behind the transfer of ascorbic acid from the blood (concentration is ∼50 *μ*M) into the cerebrospinal fluid (concentration is ∼200 *μ*M). However, very few studies are available so far in the literature utilizing ascorbic acid SVCT2 transporter for brain delivery of bioactive molecules^6^. Therefore, we designed ascorbic acid conjugated PLGA-*b*-PEG nanoparticles (NPs) for targeted brain delivery of galantamine and further evaluated them in SVCT2 expressing cell line as well as in a scopolamine induced amnetic rats. PLGA was a polymer of choice as it has already been approved by US-FDA and is completely safe. Bis amine PEG was used to provide stealth property to the nanoparticles and also to conjugate ascorbic acid. The bifunctional bis amine PEG was used to conjugate PLGA at one end and ascorbic acid at the other end.

## Results and Discussion

### Synthesis and characterization of PLGA-*b*-PEG co-polymer

PLGA-*b*-PEG co-polymer was synthesized by conjugation of PLGA-COOH with NH_2_-PEG-NH-Boc to generate PLGA-*b*-PEG-NH_2_ (Supplementary Fig. S1). The NH_2_ groups were located at both the ends of the PEG chain out of which one was free and one was blocked. The blocked NH_2_ group was deprotected from Boc after co-polymer synthesis; therefore, after NPs preparation the PEG facilitated the orientation of NH_2_ groups on the NPs surface making them available for surface chemistry. PLGA-*b*-PEG co-polymer was characterized by ^1^H NMR to confirm conjugation, and by Gel Permeation Chromatography to measure the molecular weight. The ^1^H NMR spectrum of PLGA-*b*-PEG showed ethylene oxide protons (-O-CH2*-CH2*-) of PEG at 3.65 ppm. Multiplets of PLGA at 5.2 ppm corresponds to the lactic acid (-O-CH*(CH_3_)-CO-) and 4.8 ppm corresponds to the glycolic acid (-O-CH_2_*-CO-). The Peak at 7.2 ppm attributed to amide linkage, confirming conjugation between the carboxyl group of PLGA and free amino group of PEG. Lactic repeat unit peak was also present at 1.6 ppm (Supplementary Fig. S2). ^1^H NMR results were confirmed with published reports^7–9^.

The molecular weight (M_w_) and molecular weight distribution (Mw/Mn) of co-polymers were determined by GPC. The average molecular weight of PLGA and PLGA-*b*-PEG co-polymer was 20.038 and 22.156 kDa, respectively. The M_w_/M_n_ for PLGA and PLGA-*b*-PEG co-polymer was 1.91 and 1.62, respectively. The increase in molecular weight of PLGA-*b*-PEG co-polymer evidently indicated conjugation of PLGA and PEG. The unimodal distribution also excluded the presence of free PEG. Similar results were also reported by Boddu *et al*., Danhier *et al*., and Yang *et al*.^9–11^, wherein, increase in molecular weight of PLGA was observed by GPC after conjugation of PEG.

### Preparation and optimization of PLGA-*b*-PEG NPs

PLGA-*b*-PEG NPs were prepared via nanoprecipitation method, and formulation parameters were varied to control NPs size. For controlling the NPs size and its distribution, the effect of altering the type of organic solvent (to solubilize polymer) was studied. It has been reported that the miscibility of the organic solvent with water has a remarkable impact on NPs size^12, 13^. As solvents become more miscible, the difference in solubility parameters between the solvents (∆δ) is minimized^14^. The effect of solvent miscibility with water on NPs size was investigated using two organic solvents viz. acetone and acetonitrile. Acetone poses higher water miscibility as compared to acetonitrile. As shown in Supplementary Table S1 increase in water miscibility led to a decrease in the mean NP size (polymer concentration kept constant). This is probably attributed to superior as well as efficient solvent diffusion and polymer dispersion into water. The lowest NP size obtained with acetone was 90.1 ± 5.2 nm with 0.095 ± 0.008 Pdi and 4.4 ± 0.1 mV zeta potential. Whereas, the lowest particle size attained using acetonitrile as a solvent was 107.7 ± 5.7 nm, with 0.108 ± 0.009 Pdi and 5.9 ± 0.2 mV zeta potential. A similar effect of water miscibility of organic solvent on PLGA-PEG NPs size was reported by Tang *et al*.^15^, demonstrating a decrease in particle size with increasing water miscibility. All formulations of PLGA-*b*-PEG NPs showed a positive zeta potential, which is attributed to terminal amino groups of PEG on the surface of the NPs.

The effect of altering organic to aqueous phase (solvent:water) ratio during NPs formulation was also studied with a fixed polymer concentration. In acetone formulations, NPs sizes decreased from 112.3 ± 5.7 nm to 90.1 ± 5.2 nm, as the ratio decreased from 1.0 to 0.2 (i.e. 01:01 to 01:05), respectively, attributed to better dispersibility of organic phase at 0.2 ratio (Solvent: water ratio of 01:05). However, a further decrease in ratio from 0.2 to 0.1 or increase in aqueous volume resulted in an increase in size from 90.1 ± 5.2 nm to 98.3 ± 4.9 nm. Acetonitrile formulations also showed a similar pattern where NPs sizes decreased from 137.8 ± 6.3 nm to 107.7 ± 5.7 nm, as the ratio decreased from 1.0 to 0.2, respectively. But, a further decrease in ratio from 0.2 to 0.1 or increase in aqueous volume resulted in an increase in size from 107.7 ± 5.7 nm to 110.0 ± 7.1 nm. At the solvent:water ratio of 1.0, a huge amplification in particle size was observed for both the solvents apparently due to poor phase separation^14, 16^.

A trend of increase in NPs size with an increase in polymer concentration was observed when polymer concentrations were altered during NPs synthesis at a fixed solvent:water ratio (01:05) (Supplementary Table S2). For example, NPs size increased from 88.3 ± 5.4 to 133.1 ± 7.5 nm in acetone as the polymer concentration increased from 5 to 20 mg/mL. But, increase in size from 88.3 ± 5.4 to 90.1 ± 5.2 nm was not significant (p > 0.05) when polymer concentration increased from 5 to 10 mg/mL. However, a further increase in polymer concentration from 10 to 15 resulted in significant increase in size. The sizes of the NPs were more than 100 nm at 15 mg/mL and higher polymer concentration. A similar trend was observed in acetonitrile, wherein NPs size increased from 104.6 ± 4.9 to 144.8 ± 7.1 nm as the polymer concentration increased from 5 to 20 mg/mL. The size of acetonitrile formulations were always found to be more than 100 nm. Additionally, at optimized polymer concentration, the size of acetonitrile formulation (107.7 ± 5.7 nm) was significantly higher (p < 0.05) than the size of acetone formulation (90.1 ± 5.2 nm). Zeta potential was positive for all the formulations and increased with the size of the NPs.

### Preparation and optimization of GLM loaded PLGA-*b*-PEG NPs

The optimized parameters (solvent:water ratio and polymer concentration) were used for the preparation of GLM loaded PLGA-*b*-PEG NPs (with NH_2_ functionality on the surface). Drug to polymer ratio was altered from higher to lower values i.e. 1:1, 1:2, 1:5 and 1:10 in order to optimize higher drug entrapment. The effect of the drug to polymer ratio on GLM loading is given in Supplementary Table S3.

In the case of GLM NPs prepared with acetone, decrease in the drug-to-polymer ratio from 1:1 to 1:5 (or decrease in drug amount) resulted in significant increase in the drug loading from 64.3 ± 4.2 to 81.3 ± 5.2%. This could be attributed to an adequate quantity of polymer present in the system, being sufficient to encapsulate the drug inside the polymer matrix^17^. Further decrease in the drug-to-polymer ratio from 1:5 to 1:10 resulted in significant increase in the particle size, without increasing the loading efficiency significantly. The increase of polymer might have resulted in an enhanced viscosity of organic phase. Further, this increased viscosity might cause an obstacle to the size reduction of the organic phase droplets^18^. Therefore 1:5 ratio was found to be optimum for GLM loading with minimum particle size. Similar results were obtained with GLM NPs prepared with acetonitrile. Therefore 1:5 ratio was found optimum for drug loading with a minimum particle size (Supplementary Table S3). For acetonitrile, the particle sizes were always found higher than 100 nm, which might not be suitable for brain drug delivery. In addition, at optimized drug:polymer ratio, the size of the NPs prepared using acetonitrile (135.3 ± 4.2 nm) were significantly higher (p < 0.05) than the size of the NPs prepared using acetone (95.5 ± 4.0 nm). For both solvents the size of the drug entrapped NPs was slightly higher than un-loaded NPs. The increase in size could be attributed to entrapped drug in the NP system^19^. The parameters optimized for NPs size and drug entrapment were used to synthesized GLM loaded PLGA-*b*-**mPEG** NPs (non-targeted NPs). These NPs showed entrapment efficiencies of 81.5 ± 4.9% for GLM. PLGA-*b*-**mPEG** NPs loaded with GLM showed the size of 96.2 ± 3.5 nm. Negative zeta potential obtained for the GLM loaded PLGA-*b*-**mPEG** NPs (−6.3 ± 0.3 mV) confirmed the presence of methoxy group of PEG on the surface of the NPs.

Drug loaded NPs were further characterized by differential scanning calorimetry. PLGA-*b*-PEG copolymer exhibited melting endothermic peak around 30 and 55 °C (Supplementary Fig. S3A). However, no characteristic melting peak of GLM was observed in the DSC curves of GLM-loaded PLGA-*b*-PEG NPs (Supplementary Fig. S3B). It suggests that GLM was molecularly dispersed as amorphous form in polymeric NPs matrix.

### Ascorbic acid conjugation to PLGA-*b*-PEG-NH_2_ NPs and its characterization

**Figure 1:**
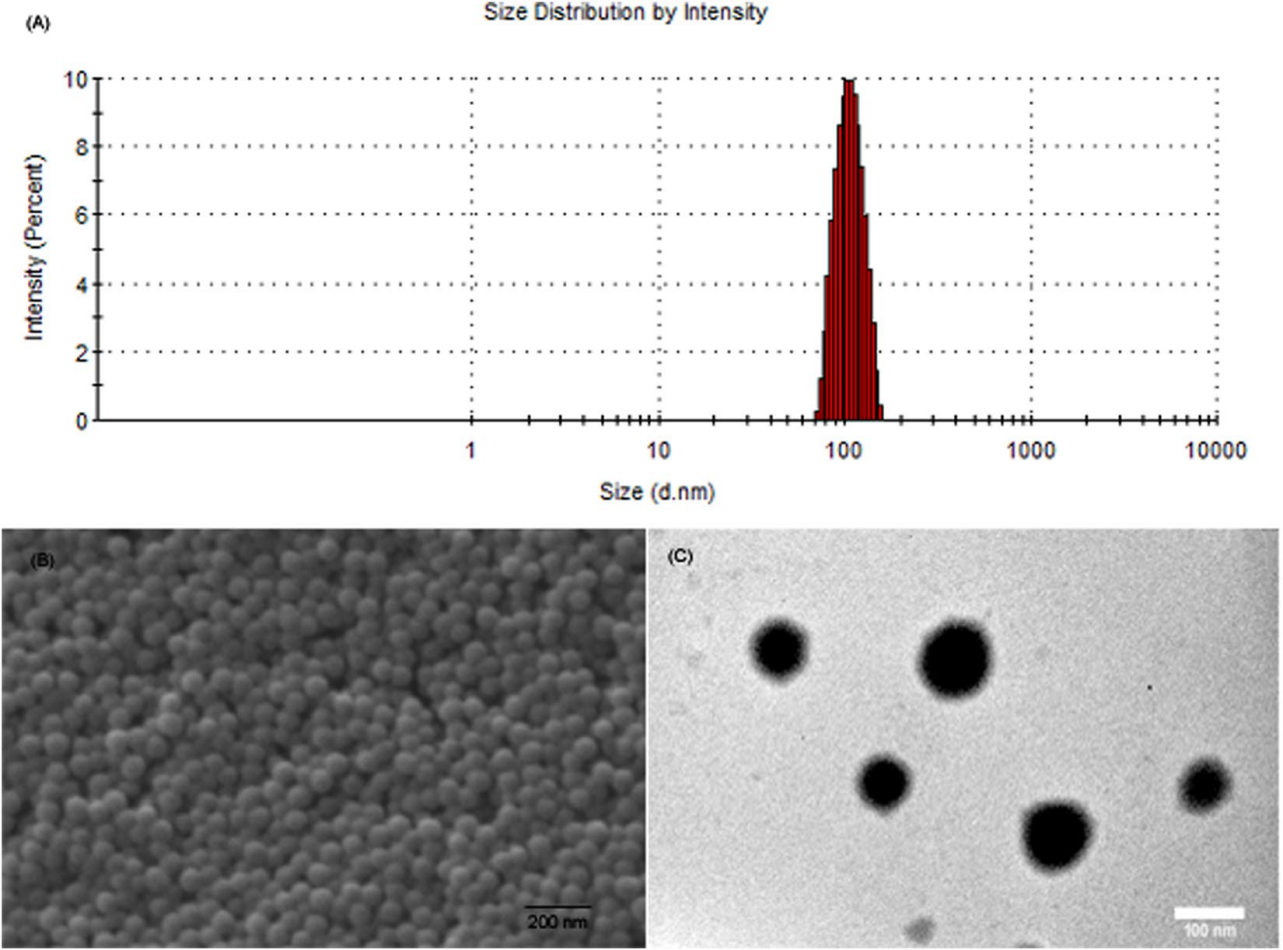
(**A**) Size distribution of the PLGA-*b*-PEG-Asc NPs as measured by DLS. (**B**) SEM image of the PLGA-*b*-PEG-Asc NPs and (**C**) TEM image of the PLGA-*b*-PEG-Asc NPs.

The optimized GLM loaded PLGA-*b*-PEG NPs formulation (using acetone as solvent) containing NH_2_ functionality on the surface were used for the conjugation of Asc (Supplementary Fig. S4). After Asc conjugation, entrapment efficiency was evaluated once again to confirm that there is no significant loss of drug during conjugation process. GLM loaded NPs showed an entrapment efficiency of 80.1 ± 3.8% after Asc conjugation as compared to entrapment efficiency of 80.7 ± 3.5% before Asc conjugation. The difference between entrapment efficiencies before and after Asc conjugation was insignificant (p > 0.05), suggesting that there was no significant loss of GLM during Asc conjugation. The PLGA-*b*-PEG-Asc NPs showed the size of 96.4 ± 3.7 nm for GLM loaded targeted NPs (Fig. 1A). The PLGA-*b*-PEG-Asc NPs showed Pdi of 0.098 ± 0.008 with zeta potential values of −7.9 ± 0.4 mV.

Interestingly, Asc conjugated NPs showed negative zeta potential as compared to positive zeta potential of parent PLGA-*b*-PEG NPs attributed to the hydroxyl group of Asc. This further confirmed conjugation of Asc on the surface of the PLGA-*b*-PEG NPs. Surface morphology of the GLM loaded PLGA-*b*-PEG-Asc was investigated using SEM and TEM. SEM photomicrograph (Fig. 1B) revealed the spherical morphology of the NPs with average particle size of about 100 nm. SEM analysis also revealed narrow, monomodal particle size distribution. TEM photomicrographs also suggested that NPs were spherical in shape and in nanometric size range (Fig. 1C). Asc conjugation on PLGA-*b*-PEG NPs was characterized by FTIR spectroscopy. For Asc conjugated PLGA-*b*-PEG NPs peaks of both Asc and NPs were observed. FTIR spectrum showed characteristic O-H stretching peak at 3523.06 cm^−1^, C-H stretching at 2900.00, C=O stretching at 1615.85, C-O stretching at 1284.81 which attributed to Asc. N-H stretching at 3328.39 and (CO)-OC stretching at 1074.09 cm^−1^ attributed to PLGA-*b*-PEG (Supplementary Fig. 5A). Additionally, the Asc conjugation was further evaluated using Thermogravimetric Analysis. About 10% higher weight loss was observed for PLGA-*b*-PEG-Asc NPs as compared to PLGA-*b*-PEG NPs, which further confirmed Asc conjugation on the surface of PLGA-*b*-PEG NPs (Supplementary Fig. 5B).

### *In vitro* drug release studies

The *in vitro* release profile of the optimized formulations of GLM loaded PLGA, PLGA-*b*-mPEG and PLGA-*b*-PEG-Asc NPs are shown in Supplementary Fig. 6. The formulations have exhibited a biphasic release pattern: one initial fast release followed by a second slow and sustained release phase. The initial burst release could be due to the release of the drug from the surface of the polymeric matrix. The drug present on the surface polymeric NPs get exposed to the release solution faster via pores and channels of the polymeric NPs and diffuses easily. Similarly, the type of polymer also affected drug release pattern. Plain PLGA NPs showed 17.8 ± 1.3% GLM release in 12 hrs. Whereas, PLGA-*b*-mPEG and PLGA-*b*-PEG-Asc NPs showed 38.2 ± 2.6 and 35.6 ± 2.9% of GLM release in 12 hrs, respectively. Higher drug release in case of PLGA-*b*-mPEG and PLGA-*b*-PEG-Asc NPs attributed to more hydrophilic nature of these co-polymers (due to the presence of PEG chains) as compared to PLGA.

The hydrophilic PEG chains allowed faster penetration of aqueous medium. After that, the GLM formulations showed a slow and sustained release pattern. The drug release was measured up to 7 days (168 hrs), and PLGA NPs exhibited only 47.1 ± 3.4% GLM release in 168 hrs, showing sustained release behaviour. The sustained behaviour resulted due to slow penetration of medium in the inner part of the NPs matrix. PLGA-*b*-mPEG and PLGA-*b*-PEG-Asc NPs showed 75.6 ± 4.6 and 73.7 ± 5.5% of GLM release in 168 hrs, respectively. The % release in 168 hrs was significantly less (p < 0.05) for plain PLGA as compared to PLGA-*b*-mPEG and PLGA-*b*-PEG-Asc, attributed to more hydrophobic nature of PLGA. Similar findings have been reported by earlier investigators who prepared core–shell nanoparticles using PLGA block co-polymer loaded with drugs^18^.

### Cellular uptake studies

**Figure 2:**
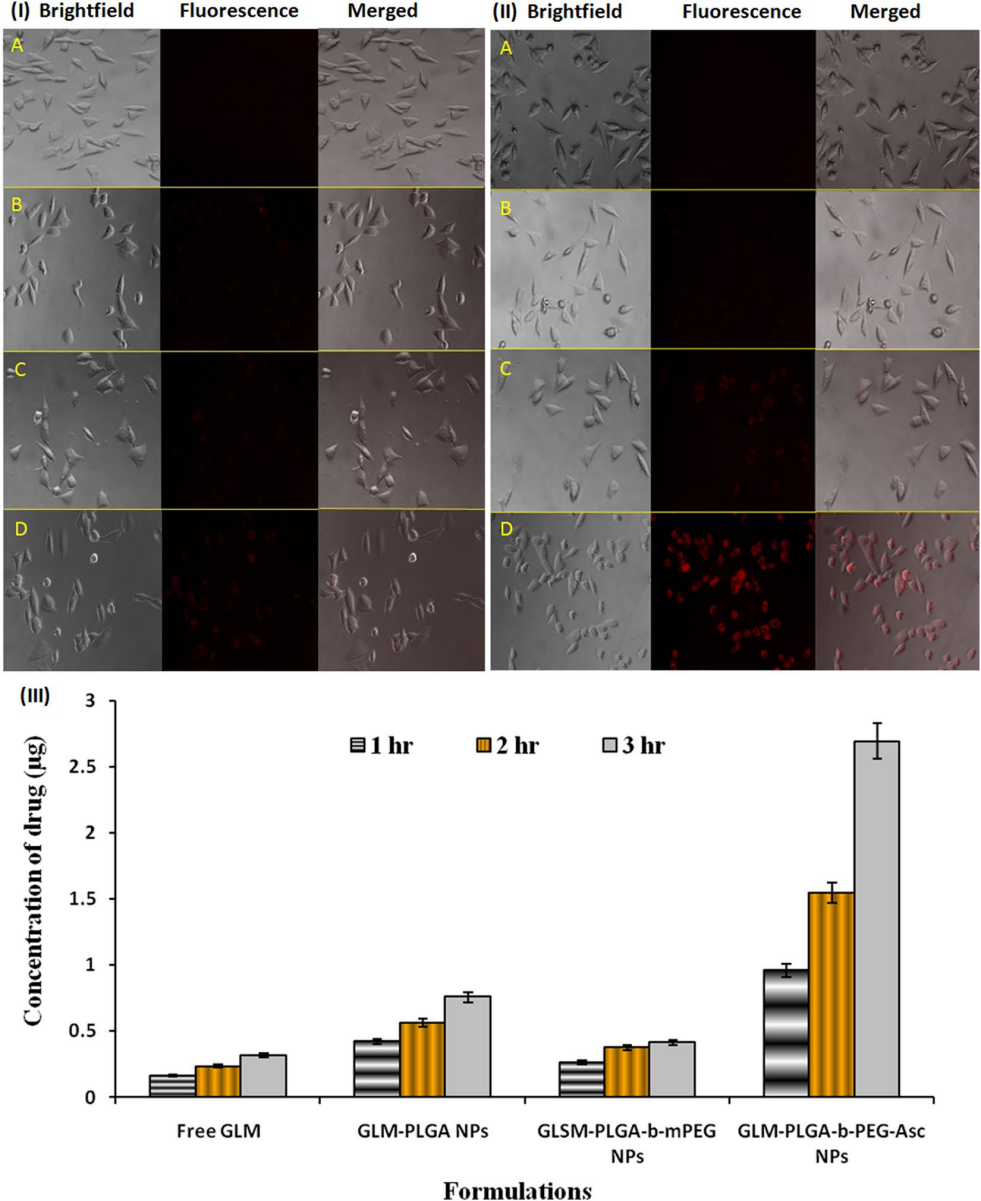
Fluorescence images for cellular uptake of different formulations after (I) 0.5 and (II) 1 hr, respectively (40X). Panel (A) free rhodamine B, Panel (B) rhodamine loaded PLGA NPs, Panel (C) rhodamine loaded PLGA-*b*-mPEG NPs and Panel (D) rhodamine loaded PLGA-*b*-PEG-Asc NPs. (III) Cellular uptake of GLM from different formulations at different time intervals as estimated by HPLC [mean ± SD (n = 3)].

Fluorescence microscopy was carried out to confirm specific uptake of Asc conjugated NPs via SVCT2 transporters present on the plasma membrane of NIT/3T3 cells (qualitatively). The results of uptake assay at 0.5 and 1 hr were shown in Fig. 2I and II, respectively. The fluorescence image of free rhodamine B showed negligible uptake by NIH/3T3 cells in 0.5 hr (Fig. 2I panel A). Plain PLGA NPs and PLGA-*b*-PEG NPs were showed very little fluorescence in 0.5 hr (Fig. 2I panel B and C). The fluorescence intensity of plain PLGA and PLGA-*b*-PEG NPs was almost same as there was no specific uptake of these NPs in the cells, indicating the lack of targeting toward this cell line. On the contrary, significantly higher fluorescence intensity was observed for PLGA-*b*-PEG-Asc (targeted) NPs in 0.5 hr (Fig. 2I panel D), attributed to the specific transportation of NPs from outer media to inside the cells.

The fluorescence images of cellular uptake at 1 hr are shown in Fig. 2II panel A to D. Fluorescence images at 1 hr further suggested negligible uptake of free rhodamine. PLGA and PLGA-*b*-PEG NPs showed slightly higher fluorescence intensity in 1 hr than 0.5 hr (Fig. 2II panel B and C). But the intensity was significantly and notably less than targeted NPs. NIH/3T3 cells incubated with rhodamine loaded PLGA-*b*-PEG-Asc (targeted) NPs showed very high fluorescence intensity at 1 hr (Fig. 2II panel D). Higher fluorescence intensity may be attributed to the specific transport of NPs from outer media to inside the cells via SVCT2 transporters present on the plasma membrane of NIH/3T3 cells^6^. Our results further confirmed that SVCT2 transporter could be utilized for the transport of Asc conjugated NPs.

Cellular uptake of free GLM and their formulations by NIH/3T3 cells has been presented in Fig. 2III. A significant increase in cellular uptake of GLM was observed for targeted NPs (PLGA-*b*-PEG-Asc), which was 8.51 times higher than that of free GLM (0.316 ± 0.027 µg; p < 0.001) at 3 hrs. The concentration of GLM for targeted NPs (2.692 ± 0.382 µg) was 3.55 and 6.53 times higher than plain PLGA (0.758 ± 0.045 µg) and PLGA-*b*-mPEG NPs (0.412 ± 0.025 µg; non-targeted), respectively at 3 hrs. Furthermore, the significantly higher concentration of GLM was estimated for targeted NPs as compared to plain PLGA and non-targeted NPs, at all time points. Increased drug concentration in cells for targeted NPs suggested a specific interaction of targeted NPs with cells^20^. The specific interaction may be attributed to Asc on the surface of the NPs and SVCT2 transporters on the plasma membrane of NIH/3T3 cells.

### *In vivo* pharmacodynamic studies

The pharmacodynamic efficacy of developed formulation was evaluated using Morris Water Maze Test, Radial Arm Maze Test and AChE activity in scopolamine induced amnetic rats.

#### Morris Water Maze Test

**Figure 3:**
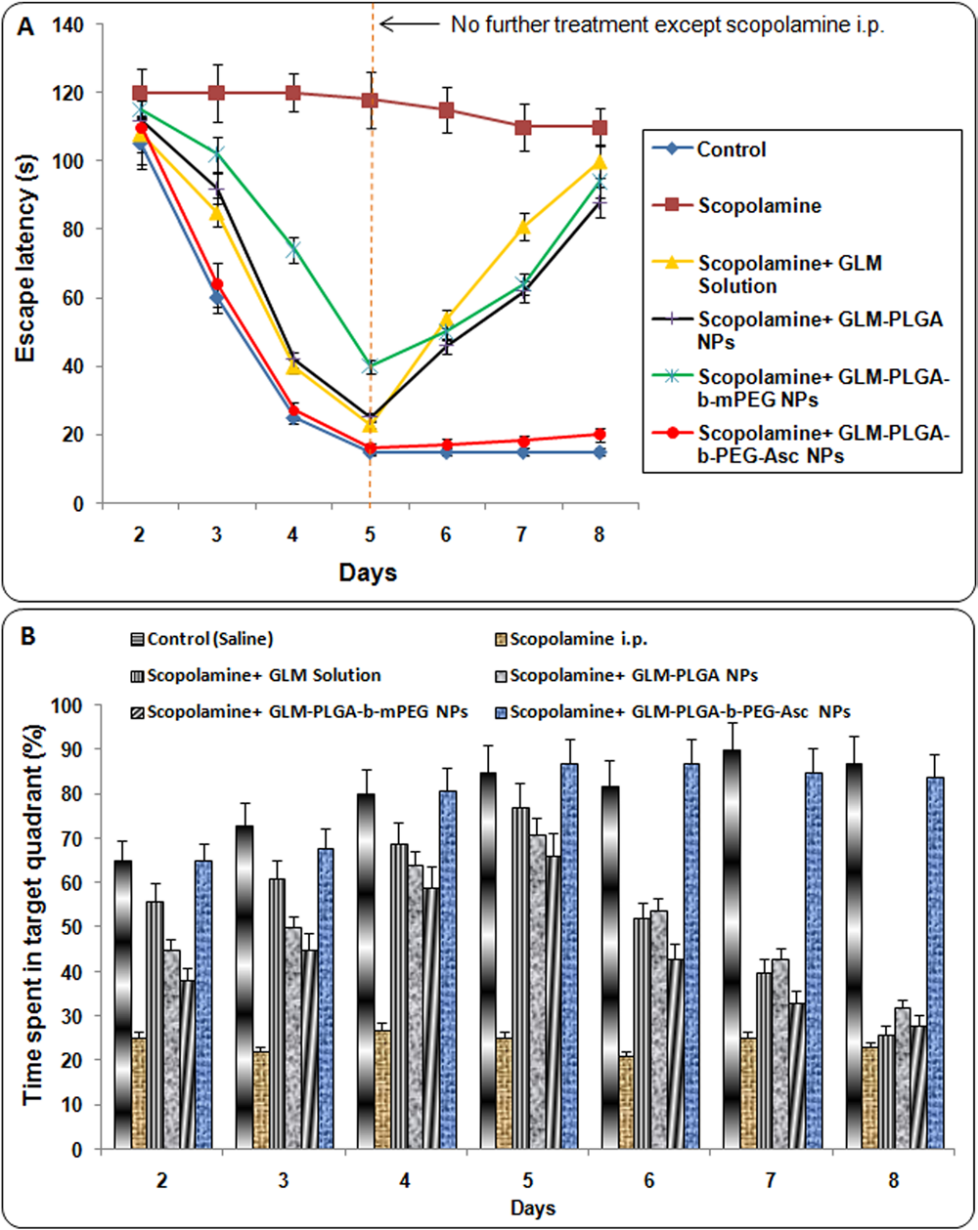
(**A**) Effect of various GLM formulations on the escape latency attained in the Morris water maze test and (**B**) Effect of various GLM formulations on time spent (%) in the target quadrant attained in the Morris water maze test.

The effects of free GLM and GLM loaded NPs on the escape latency in scopolamine treated rats are shown in Fig. 3A. The results indicated that saline treated rats quickly learned the position of the hidden platform as signified by a gradual decline in escape latency. In saline treated group, minimum escape latency was reached on day 5 and thereafter no significant decrease in escape latency was observed. In the case of scopolamine treated rats, a characteristic swimming behavior around the pool was observed, wherein rats were not able to locate the hidden platform, demonstrating loss of memory. For scopolamine treated rats escape latency period remained unchanged (no significant change) throughout the water maze test.

On the other hand, results demonstrated that administration of GLM solution and GLM NPs formulations in scopolamine treated rats significantly avert the scopolamine induced amnesia as evidenced by the significant decline in escape latency. On day 2, no significant difference in escape latencies of different formulation groups was observed which may be due to the first day of the testing phase. On subsequent days (day 3, 4 and 5; Fig. 3A) a significant decrease in escape latency was found with all GLM formulations as compared to scopolamine treated group. Same results were also observed in between the GLM formulations. The escape latency (16 ± 2 sec) for GLM-PLGA-*b*-PEG-Asc NPs (targeted NPs) was significantly (p < 0.05) lower than GLM solution (23 ± 2 sec), GLM-PLGA NPs (25 ± 3 sec) and GLM-PLGA-*b*-mPEG NPs (40 ± 3) at day 5. However, the difference was not significant (p > 0.05) as compared to GLM solution and GLM-PLGA NPs. But, the difference was very significantly lower than GLM-PLGA-*b*-mPEG NPs (p < 0.01). GLM solution and GLM-PLGA also showed significant lower escape latency (p < 0.05) than GLM-PLGA-*b*-mPEG NPs. This difference might be due to lower BBB crossing of PLGA-*b*-mPEG NPs owing to their more hydrophilic nature.

Interestingly, on the 6^th^ day (the day after stopping treatment with GLM formulations) a significant increase in escape latency was observed for GLM solution, GLM-PLGA, and GLM-PLGA-*b*-mPEG NPs as compared to GLM-PLGA-*b*-PEG-Asc NPs. In the case of GLM solution, excretion of GLM might have attributed to increase in escape latency after halting the treatment. However, insufficient BBB crossing and less accumulation in the brain were responsible for the increase in escape latency for GLM-PLGA and GLM-PLGA-*b*-mPEG NPs. On day 8, the escape latency for GLM solution group (100 ± 7 sec) was almost equal to scopolamine treated group (110 ± 6) due to complete excretion of GLM. On the other hand, GLM-PLGA-*b*-PEG-Asc group showed significant lower escape latency (20 ± 4 sec) at day 8 also as shown on day 5 (16 ± 2 sec). This may be due to the crossing of choroid plexus and higher accumulation in the brain, which resulted in the release of therapeutic amount of GLM even after stopping the NPs treatment. At day 8, the difference between escape latency of targeted NPs treated group was highly significant (p < 0.001) with all other groups and it was almost similar to control group.

For putative assessment of memory retention, the rats were subjected to a probe trial in which they swam in the pool with the platform removed. The percent time spent in platform quadrant was taken as a measure of spatial memory retention. The effect of GLM formulations on percent time spent in platform quadrant is depicted in Fig. 3B. Various groups were found to be significantly diverse regarding swimming times (%) within the target quadrant. A pattern of increase in percent time spent was observed for saline treated control group. A similar pattern was observed for GLM loaded PLGA-*b-*PEG-Asc NPs even after discontinuation of treatment with formulations after the 5^th^ day, indicating higher acetyl choline activity and retention of memory.

The shorter swimming time within the platform quadrant induced by scopolamine was significantly averted by all formulations. After day 5, a marked decrease in percent time spent was observed for free drug solution, PLGA NPs and PLGA-*b*-mPEG NPs. However, on day 6 and 7, the difference was still significantly higher for free drug solution, PLGA NPs and PLGA-*b*-mPEG NPs group than scopolamine treated group indicating sustained action. But, the percent time spent in target quadrant was almost similar on the 8^th^ day for scopolamine treated, free drug solution, PLGA NPs and PLGA-*b*-mPEG NPs group. On the contrary, PLGA-*b-*PEG-ASC NPs showed significant (p < 0.01) higher percent time spent in target quadrant on all days of testing as compared to free drugs and other NPs formulations (Fig. 3B). The results suggested that targeted drug delivery combined with the sustained action may lead to higher therapeutic benefits as compared to only sustained action.

#### Radial arm maze test

**Figure 4:**
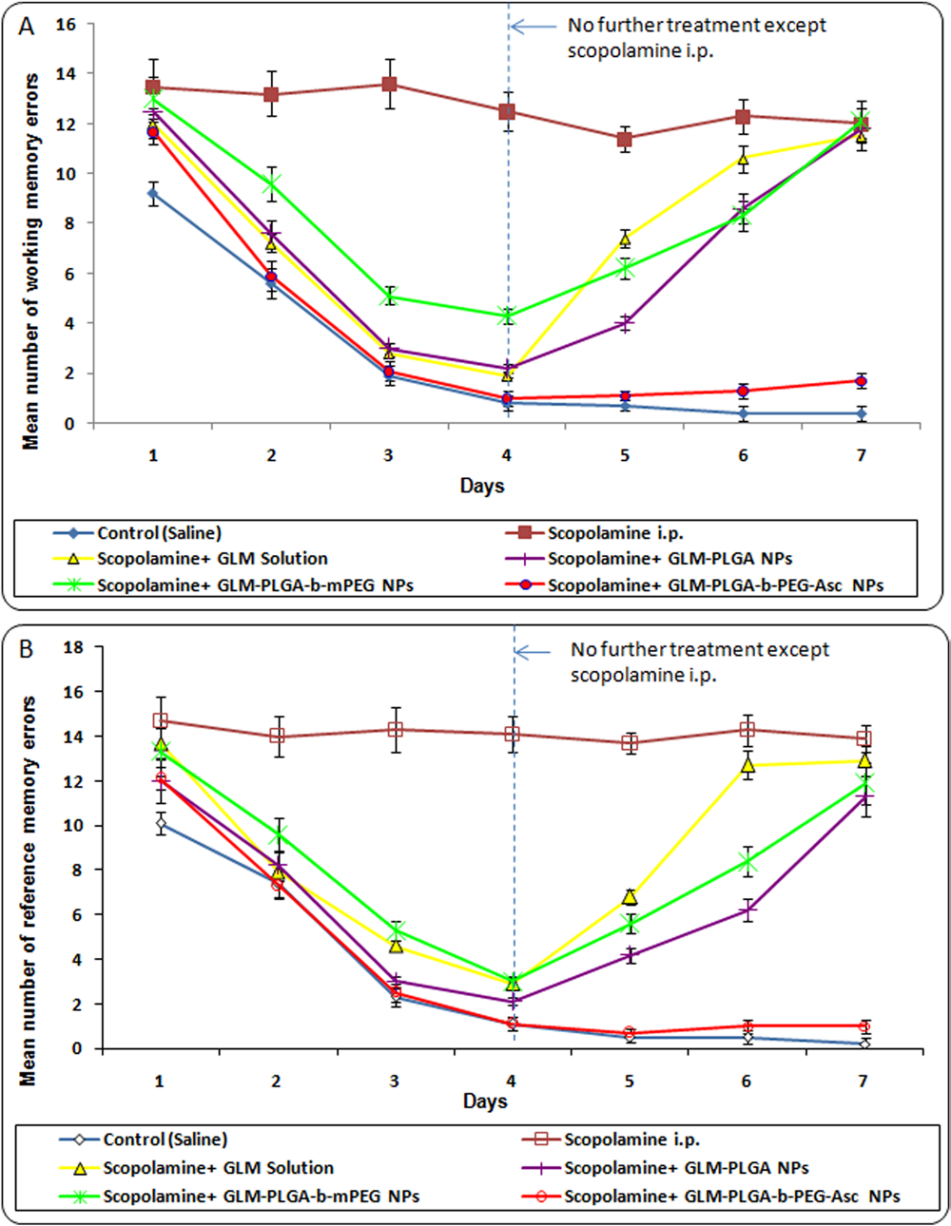
(**A**) Effect of various GLM formulations on the mean number of working memory errors achieved in the Radial arm maze test and (**B**) Effect of various GLM formulations on the mean number of reference memory errors achieved in the Radial arm maze test.

The effects of free GLM and GLM loaded NPs formulations on the working and reference memory was further evaluated by Radial arm maze test in scopolamine treated rats. The first parameter measured was working memory errors of different group (Fig. 4A). At the start of the test, all the rats initially showed poor acquisition ability, and the occurrence of reference memory errors was highest in the scopolamine treated group compared to the control group (which remained unchanged up to 7 days) indicating an induction of memory impairment. However, from day 2, a learning curve was evident in GLM solution and GLM loaded NPs formulations group, and a significant effect of treatment on the number of working memory errors was observed. The number of errors was lowest for control (saline treated) group which remained constant after day 4. Similarly, targeted NPs treated group also showed extremely significant (p < 0.001) lower number of errors as compared to other groups and was almost similar to control group (Fig. 4A).

GLM solution, GLM-PLGA NPs and GLM-PLGA*-b*-mPEG NPs also showed significantly less number of errors as compared to scopolamine treated group up to day 4. But number of errors were always found higher than targeted NPs treated group from day 2 to day 7. After day 4 (discontinuation of treatment) increase in mean number of working memory errors were observed for GLM solution, GLM-PLGA NPs and GLM-PLGA*-b*-mPEG NPs. GLM solution group showed almost similar number of working memory errors as that of scopolamine treated group on day 6 and 7. However, GLM-PLGA and GLM-PLGA-*b*-mPEG NPs still showed significant (p < 0.05) less number of errors on day 6 than scopolamine treated and GLM solution treated group, which became almost similar on day 7 (Fig. 4A). This may be attributed to sustained action exerted by NPs formulations.

GLM formulations were further assessed on the basis of reference memory errors i.e. entry to an empty arm, and the results are depicted in Fig. 4B. Again, scopolamine treated group showed the highest number of reference memory errors. All other formulations followed a similar pattern as that of working memory errors. Targeted NPs treated group showed significantly (p < 0.001) lower number of reference memory errors than other groups and was almost similar to control (saline treated) group. The sustained action was also similar to that of working memory test.

**Figure 5:**
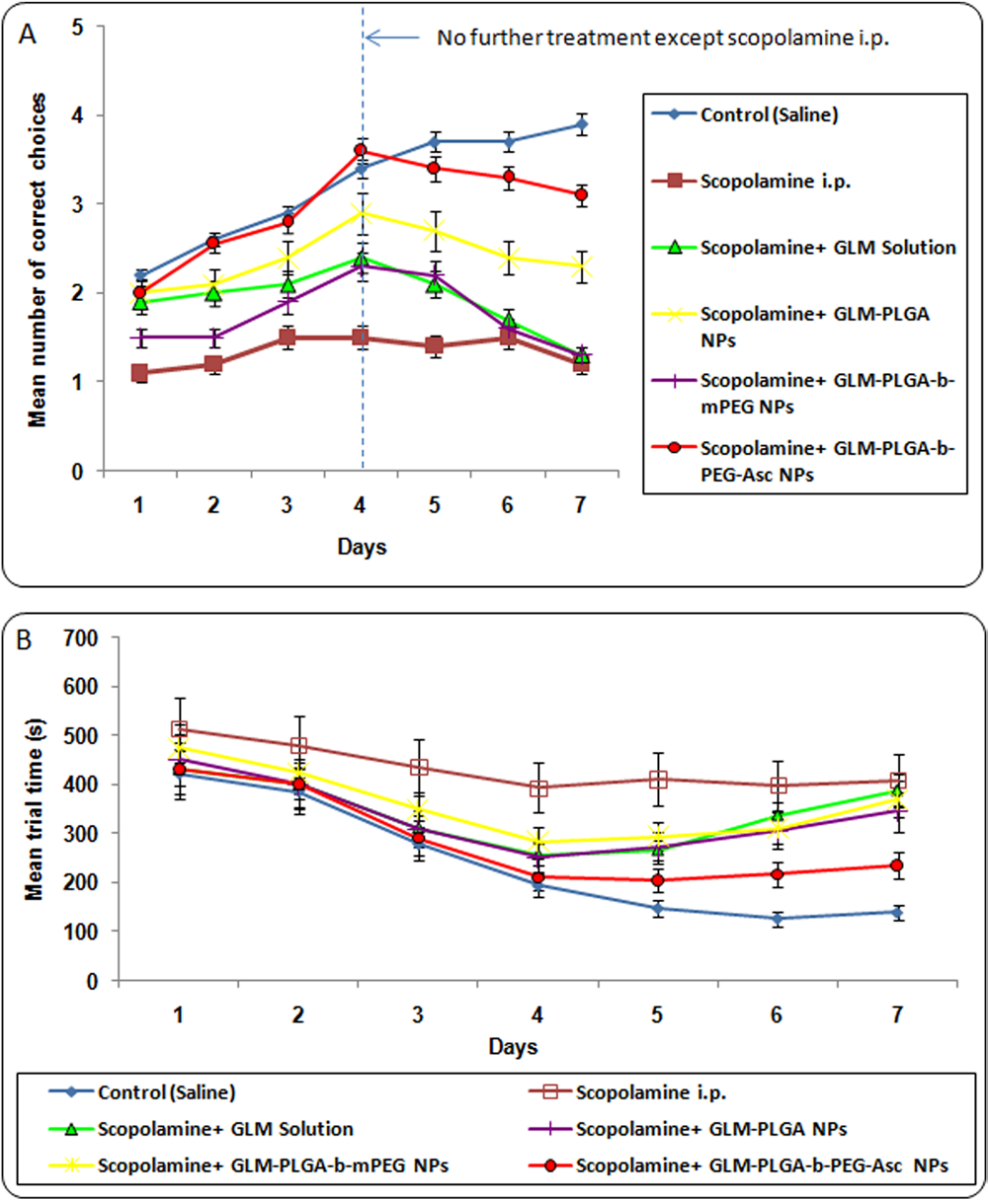
(**A**) Effect of various GLM formulations on the mean number of correct choices achieved in the Radial arm maze test and (**B**) Mean trial time for various GLM formulations achieved in the Radial arm maze test.

Number of correct choices was the third parameter assessed by radial arm maze test, and the results for GLM formulations are shown in Fig. 4B. A learning curve was evident from the results, and a significant effect of treatment with free drug and drug loaded NPs on the number of correct choices was observed during the learning process. The number of correct choices was significantly lower (p < 0.05) in scopolamine treated groups than control and GLM formulations treated group. The scopolamine treated group showed not only a decreased number of correct choices but also a significantly higher (p < 0.05) mean trial time was observed. On the contrary GLM formulations treated groups showed significantly improved acquisition ability of spatial memory task. Inter-group comparison of number of correct choices and mean trial time demonstrated that drug loaded PLGA-*b*-PEG-Asc NPs showed significantly higher (p < 0.05) number of correct choices and significantly lower (p < 0.05) mean trial time than drug solution, PLGA NPs and PLGA-*b*-mPEG NPs (Fig. 5A and B). Higher brain accumulation and subsequent sustained action were also evident in the case of targeted NPs. On the other hand, drug solution, PLGA NPs and PLGA-*b*-mPEG NPs showed less sustained effect and reaches to the brain cells at lesser extent as that of targeted NPs. The higher therapeutic outcome might be resulted due to the transport of Asc conjugated NPs through SVCT2 transporters^7, 21, 22^.

#### Activity of acetyl cholinesterase (AChE)

**Figure 6:**
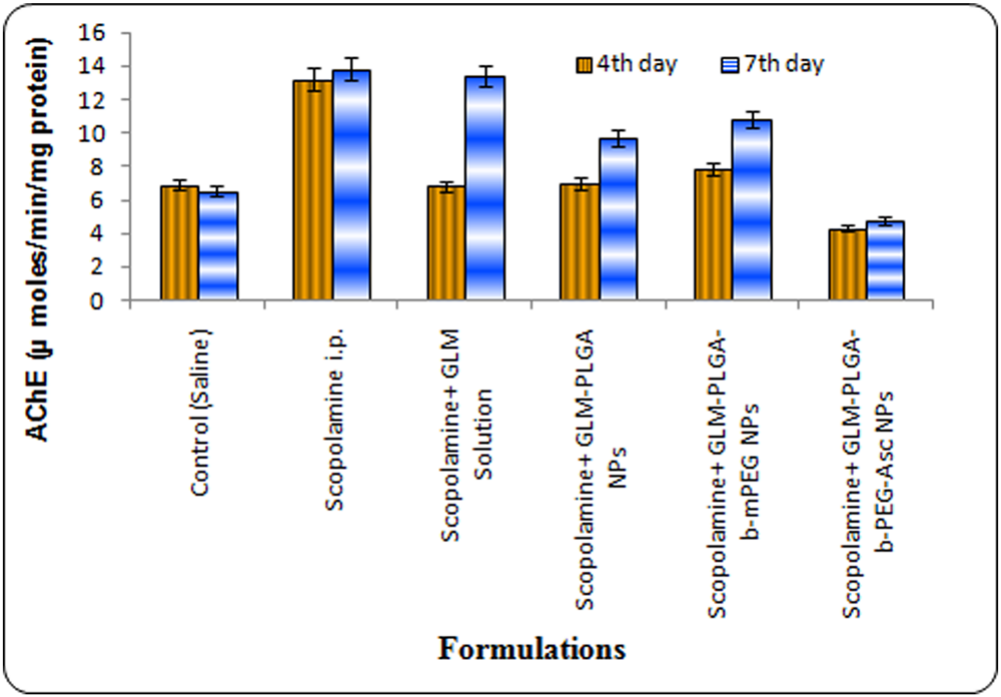
AChE levels at 4^th^ and 7^th^ day after treatment with different GLM formulations.

The results of AChE activity at day 4 and 7 are shown in Fig. 6. Scopolamine treated group showed extremely significant higher (p < 0.001) AChE activity than other groups. The AChE activity of the whole brain was distinctly reduced (p < 0.001) in GLM treated groups, which is considered as a sign of inhibition of AChE activity in rat brain. The lowest AChE activity on day 4 was found for drug loaded PLGA-*b*-PEG-Asc NPs treated group (4.3 ± 0.2 µmoles/min/mg protein for GLM loaded targeted NPs). The lowest AChE activity on day 7 was also observed for PLGA-*b*-PEG-Asc NPs treated group (4.7 ± 0.4 µmoles/min/mg protein for GLM loaded targeted NPs).

Interestingly, AChE activity at 4^th^ and 7^th^ day in targeted NPs treated group was found lower than control (saline treated) group. Drug loaded PLGA and PLGA-*b*-mPEG NPs also showed significantly lower AChE activity than scopolamine treated group at day 4. However, AChE activity was found to be increased at day 7 for these groups after discontinuation of treatment. This may be due to excretion of drug and insufficient drug available for action. Free drug solution group showed almost similar AChE activity to that of scopolamine treated group at day 7 (Fig. 6). The results were in accordance with the previous reports wherein the drug treated groups showed significant lower AChE activity than scopolamine treated group^23–25^.

### Biodistribution studies

The *in vivo* brain targeting efficiency of Asc conjugated NPs was further evaluated via biodistribution studies. Biodistribution of the drug via free GLM solutions and GLM loaded NP formulations (GLM-PLGA, GLM-PLGA-*b*-mPEG, GLM-PLGA-*b*-PEG-Asc) were assessed. The concentration of GLM in different organs was estimated via HPLC at 1^st^, 6^th^, and 24^th^ hr. The GLM-PLGA-*b*-PEG-Asc NPs have shown extremely significant (p < 0.001) higher accumulation of GLM in the brain as compared to GLM-PLGA, GLM-PLGA-*b*-mPEG and free GLM (Table 1). The GLM-PLGA-*b*-PEG-Asc NPs have shown approximately 4.3, 3.2 and 3.9 times higher penetration of GLM in the brain as compared to free GLM, GLM-PLGA NPs and GLM-PLGA-*b*-mPEG NPs, respectively in 1 hr.

**Table 1:** Biodistribution of GLM in different organs.

Organ	Formulation	Concentration (ng/g tissue)		
		1 hr	6 hr	24 hr
Brain	GLM solution	62.5 ± 5.3	103.6 ± 7.7	46.9 ± 3.1
	GLM-PLGA	*84.5 ± 6.8	***248.9 ± 18.6	***317.4 ± 18.4
	GLM-PLGA-*b*-mPEG	*69.8 ± 4.2	***216.5 ± 14.7	***268.9 ± 13.7
	GLM-PLGA-*b*-PEG-Asc	***272.8 ± 16.4	***746.1 ± 30.9	***893.7 ± 54.2
Liver	GLM solution	563.3 ± 26.3	1928.6 ± 136.4	1067.4 ± 86.3
	GLM-PLGA	615.3 ± 36.4	1279.3 ± 93.5	1858.4 ± 146.7
	GLM-PLGA-*b*-mPEG	369.2 ± 21.8	603.4 ± 45.2	962.7 ± 51.3
	GLM-PLGA-*b*-PEG-Asc	387.4 ± 24.5	621.5 ± 44.2	806.2 ± 46.5
Spleen	GLM solution	452.6 ± 25.4	689.3 ± 47.2	934.5 ± 52.4
	GLM-PLGA	589.3 ± 37.8	729.7 ± 45.1	1105.2 ± 84.6
	GLM-PLGA-*b*-mPEG	337.5 ± 17.8	536.1 ± 25.5	761.5 ± 73.1
	GLM-PLGA-*b*-PEG-Asc	275.3 ± 21.5	490.5 ± 32.7	658.2 ± 32.7
Lung	GLM solution	385.6 ± 28.5	749.5 ± 57.3	681.3 ± 46.4
	GLM-PLGA	736.8 ± 40.2	1248.3 ± 103.3	965.3 ± 83.5
	GLM-PLGA-*b*-mPEG	347.2 ± 27.1	583.4 ± 39.1	817.2 ± 60.3
	GLM-PLGA-*b*-PEG-Asc	331.5 ± 23.6	536.1 ± 28.4	734.9 ± 31.5
Kidney	GLM solution	593.6 ± 59.3	1042.6 ± 87.3	621.7 ± 37.5
	GLM-PLGA	379.3 ± 26.5	559.3 ± 41.4	953.2 ± 44.8
	GLM-PLGA-*b*-mPEG	269.5 ± 24.3	403.1 ± 27.4	702.4 ± 52.4
	GLM-PLGA-*b*-PEG-Asc	295.6 ± 21.6	436.4 ± 20.8	638.1 ± 33.5

*p < 0.05, significant; ***p < 0.001, extremely significant.

Values represented as mean ± SD (n = 3).

Similarly, at 6^th^ hr, the GLM-PLGA-*b*-PEG-Asc NPs have shown approximately 7.2, 2.9 and 3.4 times higher penetration of GLM in the brain as compared to free GLM, GLM-PLGA NPs, and GLM-PLGA-*b*-mPEG NPs, respectively. Furthermore, the concentration was approximately 19, 2.8 and 3.3 times higher than free GLM, GLM-PLGA NPs, and GLM-PLGA-*b*-mPEG NPs, respectively at 24 hr. Higher concentration of GLM in the brain via PLGA-*b*-PEG-Asc NPs clearly suggested that Asc conjugation facilitated transport of targeted NPs. Gajbhiye *et al*.^26^ and Wilson *et al*.^22^, also reported higher brain uptake of drugs via targeted nanocarriers.

The distribution of GLM in the brain was continuously increased from 1^st^ to 24^th^ hr in the case of PLGA, PLGA-*b*-mPEG, and PLGA-*b*-PEG-Asc NPs, while free GLM have shown an increase in concentration from 1^st^ to 6^th^ hr and then decreased at 24^th^ hr. This decrease may be attributed to excretion of GLM from the body. However, extremely significant higher accumulation of GLM in the brain via targeted NPs resulted not only in higher therapeutic benefit but also a significantly sustained therapeutic action (Table 1). Higher concentration of GLM in the brain via PLGA-*b*-PEG-Asc NPs further confirm that Asc conjugation facilitated transport and accumulation of targeted NPs in the brain.

Free GLM has shown higher accumulation in metabolizing organ e.g. liver than NPs formulations, which was found to increase from 1^st^ to 6^th^ hr and then decreased at 24^th^ hr. The metabolism of GLM takes place in the liver, which attributed to its accumulation in the liver. Non-specific interaction resulted in the significant higher accumulation of GLM in liver, lung, and spleen via plain PLGA NPs. However, significant less accumulation in liver, spleen, lungs, and kidneys was observed in the case of PEGylated NPs (PLGA-*b*-mPEG and PLGA-*b*-PEG-Asc). Additionally, it was clearly evident that targeting as well as PEGylation (PLGA-*b*-PEG-Asc NPs) were considerably useful for the delivery of drug to the brain to achieve the higher therapeutic outcome.

In conclusion, this study reports the potential of PLGA-*b*-PEG-Asc for brain delivery of galantamine. *In vitro* results suggested that Asc conjugation can enhance cellular uptake of NPs in SVCT2 transporters expressing cells. The *in vivo* results were analogous to *in vitro* cell line results. Pharmacodynamic studies (Morris water maze test, Radial arm test, and AChE activity) demonstrated higher therapeutic outcome via Asc conjugated NP formulations than plain PLGA NPs, PLGA-*b*-mPEG NPs and free drugs. Biodistribution studies further corroborated the Morris water maze test, Radial arm test, and AChE activity, wherein highest concentrations of drugs were assessed for targeted NPs in the brain. Biodistribution studies further evidence that PEGylated and surface anchored NPs reduced the accumulation of drugs in excretory organs than free drug and plain PLGA NPs. The developed targeted NPs have shown the potential to cross the choroid plexus significantly and can deliver the significantly higher amount of drug to the brain than free drug, plain PLGA and PLGA-*b*-mPEG NPs for the higher therapeutic outcome. Apart from anti-Alzheimer’s drugs, other bioactives can also be delivered efficiently using Asc anchored NPs for different ailments of the brain. Thus, it can be concluded that GLM loaded Asc conjugated PLGA-*b*-PEG NPs hold strong potential to treat Alzheimer’s, with fewer side effects.

## Methods

Detailed procedure for (a) Synthesis and characterization of PLGA-*b*-PEG co-polymer, (b) Preparation and optimization of PLGA-*b*-PEG NPs, (c) Preparation and optimization of GLM loaded PLGA-*b*-PEG NPs, (d) Ascorbic acid conjugation to PLGA-*b*-PEG-NH_2_ NPs and its characterization, (e) *In vitro* drug release studies, (f) Cellular internalization studies in SVCT2 expressing cell line and (g) Biodistribution studies are given in supplementary information.

### *In vivo* pharmacodynamic studies

The pharmacodynamic potential of developed targeted formulations was compared with free drugs and plain NPs formulations using scopolamine induced amnesia in albino rats (Sprague Dawley strain, 7–8 weeks old weighing 200 ± 20 g). All the animal experiments were performed according to the protocol approved by the Institutional Animal Ethical Committee of Dr. Hari Singh Gour Central University, Sagar (MP), India (registration no. 379/01/ab/CPCSEA; 13/828). The pharmacodynamic studies were performed using Morris Water Maze Test and Radial Arm Maze Test.

#### Morris Water Maze Test

To assess the potential of targeted NPs on reference memory capacities, Morris water maze test was carried out as reported in the literature with slight modification^27–30^. The Maze test consisted of training and testing session. Before the test, rats were housed in controlled condition (12 hrs light/12 hrs dark schedule; 25 ± 1 °C). Food and water were provided *ad libitum*. The water maze apparatus consisted of a rounded pool of 120 cm in diameter and 45 cm in height^30, 31^ with a featureless inner surface (Supplementary Fig. S7A). The pool was divided into 4 equal quadrants containing a transparent column platform of 10 cm diameter in one quadrant. Each quadrant was provided with a clue (hint or sign). The pool was filled to a height of 25 cm with water (25 ± 1 °C), and the height of the platform (23 cm) was adjusted such that it was submerged 2 cm below the water surface (hidden platform). The platform was situated in the pool away from the pool wall. The non-toxic white paint was added to the water to make it opaque^28^. Location of the pool and platform was fixed during the entire experiment period.

#### Experimental protocol

Flow chart of the experimental protocol for water maze test is given in Supplementary Fig. S7B. Animals were divided into 6 groups each containing 6 animals (n = 6). The first experimental day was dedicated to swimming training in the absence of the platform. During the subsequent days, the rats were given four training-trials sessions per day to locate the platform (with the platform in place). The inter training trial interval of 5 min was kept. During this learning phase, rats were placed randomly in the pool at selected locations and allowed to search the platform. The time taken from starting point to the escape from water onto the hidden platform (escape latency; learning and reference memory test {1^st^ test}) was measured in each training session. Each trial was ended if the animal arrived at the platform or after 120 s, whichever came first. Animal failing to arrive at the platform in 120 s were guided onto it. The rat was allowed to remain on the platform for 30 s after each hidden-platform trial. The location of the platform was fixed during the entire test period.A probe trial session was carried out on each day after 2 hr of last escape latency test, wherein platform was detached from the pool and rat were allowed to explore for the platform for 120 s. The time spent in the target quadrant was considered as a measure of spatial memory retention (2^nd^ test).

#### Treatment

Animals were divided into 6 groups each containing 6 animals (n = 6). All the treatment were started from the 2^nd^ day of training as the 1^st^ day was dedicated to swimming training. Amnesia was induced in all groups except group 1 by intraperitoneal (i.p.) injection of scopolamine hydrochloride (0.4 mg/kg of body weight) in 0.9% normal saline 30 min prior to the training session (once a day)^21, 31^. Group 1 was injected with normal saline and treated as normal control. Group 2 was injected with scopolamine only (disease control) and didn’t receive any formulation. All other groups were administered with different formulations intravenously via tail vein according to grouping on 2^nd^, 3^rd^, 4^th^ and 5^th^ day of treatment. The escape latency and probe test were done from day 2 to day 8. However, no treatment except scopolamine was given on the 6^th^, 7^th^ and 8^th^ day. Testing on the 6^th^, 7^th^ and 8^th^ day was carried out to evaluate any sustained effect exerted by the formulation.

The details of different formulations are as follows: group 3 = GLM solution (GLM), group 4 = GLM loaded plain PLGA NPs (GLM-PLGA), group 5 = GLM loaded PLGA-*b*-mPEG NPs (GLM-PLGA-*b*-mPEG), group 6 = GLM loaded PLGA-*b*-PEG-Asc NPs (GLM-PLGA-*b*-PEG-Asc) (1.5 mg/kg equivalent of GLM). NPs formulations were suspended in saline and injected intravenously via tail vein 1 hr before testing (30 min before scopolamine treatment)^21, 31^.

#### Radial arm maze test

The radial arm maze was designed for evaluating spatial memory retention in the rats. The maze had eight arms radiating from a central platform. A small food cup was placed at the end of each arm (Supplementary Fig. S7C). The design of the maze ensures that, after eating the food of one arm, the rat has to return to the central platform before going to the next arm. Each arm was 50 cm in length and 10 cm wide with a 30 cm central platform^32^. Various extra-maze cues were placed around the maze, and the position of maze and cues were remained same until experiments were complete.

#### Training phase

Before training, the rats were kept on a restricted diet to reduce their body weights up to 85–90% of normal weight over a 2 week period to motivate the rats to seek food in the maze^24, 33^. While the water was freely available to them. The maze test consisted of a 7 day training session and a 7 day testing session (Supplementary Fig. S7C). On day 1 of the training session, rats were given 15 min to adapt to the maze arms without any food in the food cups. From days 2 to 7 of the training session, rats received 10 min trials twice a day, separated by an interval of 5 min. During the training, food palette was placed in all cups of eight arms. The rat was placed on the central platform of the maze and was allowed to explore food freely in all arms until 10 min.

#### Testing session

During the testing session, food pallets were placed in four cups of four selected arms and other four arms were kept empty^33^. The location of food and extra-maze cues were not changed during the experiments. At the beginning of each testing trial, the rat was placed on the central platform and was allowed to explore for the food. The trial was continued until rat consumed all four food pellets or until 10 min had elapsed. Performance parameters that were recorded during testing trials included number of working memory errors (re-entry to earlier visited arm where food was already consumed), the number of reference memory errors (entry to an empty arm) and the number of correct choices (entry to a correct arm to consume food). Time taken to consume all four food pallets (Mean trial time) was also recorded.

#### Treatment

Animals were divided into 6 groups each containing 6 animals, and similar treatment regimen was followed as carried out in water maze test. The details of different formulations are also same as mentioned in water maze test. The performance parameters were recorded from day 1 to day 7 of the testing phase.

#### Activity of acetyl cholinesterase (AChE)

AChE activity was assessed by the earlier reported method^24, 30^. Three animals from each group of the radial maze test were sacrificed on 4^th^ Day and remaining three animals were sacrificed immediately after the last radial maze test (7^th^ Day) by decapitation. The whole brain was removed and homogenized in sodium phosphate buffer (75 mM, pH 7.4, 4 °C) separately. For the assay of AChE activity, 0.1–0.2 ml homogenate was incubated (37 °C, 20 min) with a 4 ml reaction mixture containing acetylthiocholine iodide (0.3 mM) and 1 ml sodium phosphate buffer (0.1 mM, pH7.4). The reaction was ended by addition of 3% sodium lauryl sulfate (1 mL). Subsequently, 1 ml of 0.2% 5,5′-dithiobis(2-nitrobenzoic acid) was added to give the yellow anion of the 5-thio-2-nitrobenzoic acid. The colour intensity was observed spectrophotometrically at 440 nm, and AChE activity was measured as optical density (OD) value/mg protein for AChE.

### Statistical analysis

The statistical analysis was performed with Graph Pad Instat Software (Version 3.00, Graph Pad Software, San Diego, California, USA) using one-way ANOVA followed by Tukey–Kramer multiple comparison tests. The difference with p < 0.05 was considered statistically significant.

## Electronic supplementary material

Revised Supplementary Information

## References

[1] Nunes-Tavares N (2012). Inhibition of choline acetyltransferase as a mechanism for cholinergic dysfunction induced by amyloid-β peptide oligomers. J. Biol. Chem..

[2] Lockhart IA, Mitchell SA, Kelly S (2009). Safety and tolerability of donepezil, rivastigmine and galantamine for patients with Alzheimer’s disease: systematic review of the ‘real-world’ evidence. Dement. Geriatr. Cogn. Disord..

[3] Hampel DH, Pantel J (2010). Galantamine for Alzheimer’s disease. Expert Opin. Drug Metab. Toxicol..

[4] Gajbhiye KR, Gajbhiye V, Soni V (2015). Targeted brain delivery of bioactive molecules using nanocarriers. J. Bioequiv. Availab..

[5] Rice ME (2000). Ascorbate regulation and its neuroprotective role in brain. Trends Neurosci..

[6] Salmaso S (2009). Targeting clioma cells *in vitro* with ascorbate-conjugated pharmaceutical nanocarriers. Bioconjugate Chem..

[7] Chen J, Li S, Shen Q (2012). Folic acid and cell-penetrating peptide conjugated PLGA–PEG bifunctional nanoparticles for vincristine sulfate delivery. Euro. J. Pharm. Sci..

[8] Lin G, Cosimbescu L, Karin NJ, Tarasevich BJ (2012). Injectable and thermosensitive PLGA-g-PEG hydrogels containing hydroxyapatite: preparation, characterization and *in vitro* release behaviour. Biomed Mater..

[9] Boddu SHS (2012). Preparation and characterization of folate conjugated nanoparticles of doxorubicin using PLGA-PEG-FOL polymer. Med. Chem..

[10] Danhier F (2009). Targeting of tumor endothelium by RGD-grafted PLGA-nanoparticles loaded with Paclitaxel. J. Control. Release..

[11] Yang J (2007). Antibody conjugated magnetic PLGA nanoparticles for diagnosis and treatment of breast cancer. J. Mater Chem..

[12] Galindo-Rodriguez S, Allemann E, Fessi H, Doelker E (2004). Physicochemical parameters associated with nanoparticle formation in the salting-out, emulsification-diffusion, and nanoprecipitation methods. Pharm. Res..

[13] Bilati U, Allemann E, Doelker E (2005). Development of a nanoprecipitation method intended for the entrapment of hydrophilic drugs into nanoparticles. Eur. J. Pharm. Sci..

[14] Cheng J (2007). Formulation of functionalized PLGA–PEG nanoparticles for *in vivo* targeted drug delivery. Biomaterials.

[15] Tang, L. *et al*. Immunosuppressive activity of size-controlled PEG-PLGA nanoparticles containing encapsulated cyclosporine A. *J*. *Transplant*. 89614 (2012). 10.1155/2012/896141 PMC3321582 22545201

[16] Dhankar R (2011). HER-2 targeted immunonanoparticles for breast cancer chemotherapy. J. App. Pharm. Sci..

[17] Afshari M, Derakhshandeh K, Hosseinzadeh L (2014). Characterisation, cytotoxicity and apoptosis studies of methotrexate-loaded PLGA and PLGA-PEG nanoparticles. J. Microencapsul..

[18] Pagar K, Vavia P (2013). Rivastigmine-loaded l-lactide-depsipeptide polymeric nanoparticles: decisive formulation variable optimization. Sci. Pharma..

[19] Cooper DL, Harirforoosh S (2014). Design and optimization of PLGA-based diclofenac loaded nanoparticles. PLos One.

[20] Li L (2014). Epithelial cell adhesion molecule aptamer functionalized PLGA-lecithin-curcumin-PEG nanoparticles for targeted drug delivery to human colorectal adenocarcinoma cells. Int. J. Nanomedicine.

[21] Joshi SA, Chavhan SS, Sawant KK (2010). Rivastigmine loaded PLGA and PBCA nanoparticles: preparation, optimization, characterization, *in vitro* and pharmacodynamic studies. Euro. J. Pharm. Biopharm..

[22] Wilson B (2008). Poly(n-butylcyanoacrylate) nanoparticles coated with polysorbate 80 for the targeted delivery of rivastigmine into the brain to treat Alzheimer’s disease. Brain Res..

[23] Pena ID (2014). Effects of ginseol k-g3, an Rg3-enriched fraction, on scopolamine-induced memory impairment and learning deficit in mice. J. Ginseng Res..

[24] Bastiat G (2010). Tyrosine-based rivastigmine loaded organogels in the treatment of Alzheimer’s disease. Biomaterials.

[25] Kulkarni KS, Kasture SB, Mengi SA (2010). Efficacy study of Prunus amygdalus (almond) nuts in scopolamine-induced amnesia in rats. Ind. J. Pharmacol..

[26] Gajbhiye V, Jain NK (2011). The treatment of Glioblastoma Xenografts by surfactant conjugated dendritic nanoconjugates. Biomaterials.

[27] Yang MH (2009). Neuroprotective effects of *Dioscorea opposita* on scopolamine-induced memory impairment in *in vivo* behavioral tests and *in vitro* assays. J. Ethnopharmacol..

[28] Ricobaraza A (2009). Phenylbutyrate Ameliorates Cognitive Deficit and Reduces Tau Pathology in an Alzheimer’s Disease Mouse Model. Neuropsychopharmacol..

[29] Zamani Z, Reisi P, Alaei H, Pilehvarian AA (2012). Effect of Royal Jelly on spatial learning and memory in rat model of streptozotocin-induced sporadic Alzheimer’s disease. Adv. Biomed. Res..

[30] Zhang P (2007). *In vitro* and *in vivo* evaluation of donepezil-sustained release microparticles for the treatment of Alzheimer’s disease. Biomaterials.

[31] Jogani VV, Shah PJ, Mishra P, Mishra AK, Misra AR (2008). Intranasal mucoadhesive microemulsion of tacrine to improve brain targeting. Alzheimer Dis. Assoc. Disord..

[32] Tanabe F, Miyasaka N, Kubota T, Aso T (2004). Estrogen and progesterone improve scopolamine-induced impairment of spatial memory. J Med Dent Sci..

[33] Cheng KK (2013). Highly stabilized curcumin nanoparticles teted in an *in vitro* blood-brain model and in Alzheimer’s disease Tg2576 mice. The AAPS J..

