# Supplementary material for: Ascorbic acid tethered polymeric nanoparticles enable efficient brain delivery of galantamine: An in vitro-in vivo study
Source: Sci Rep. 2017 Sep 11;7:11086. doi: 10.1038/s41598-017-11611-4 (PMC5594022; doi:10.1038/s41598-017-11611-4)
Supplement: Supplementary file 1 — Revised Supplementary Information [file 41598_2017_11611_MOESM1_ESM.pdf]

**Supplementary Information**

# Ascorbic acid tethered polymeric nanoparticles enable efficient brain delivery of galantamine: An *in vitro-in vivo* study

Kavita R. Gajbhiye, Virendra Gajbhiye, Imtiaz A. Siddiqui, Srikanth Pilla, Vandana Soni*

## Materials and Methods

### Materials

Free carboxylic acid terminated PLGA (PLGA-COOH; lactide/glycolide ratio of 50:50, Mol. wt. 20 kDa) was obtained from Sun Pharma Advanced Research Company Ltd., Baroda (India) as a benevolent gift. Bis amine polyethylene glycol [$NH_2$-PEG-NH-Boc (One amine group protected via butyloxycarbonyl {Boc}), Mol. wt. 2000 Da] and methoxy-PEG amine (mPEG-$NH_2$; Mol. wt. 2000 Da) were purchased from JenKem Technology (USA). Galantamine HBr (GLM) was obtained as a gift sample from M/s Cadila Pharma. Ltd., India. Ascorbic acid, N-hydroxysuccinimide (NHS), -ethyl-3-(3-dimethylaminopropyl)-carbodiimide (EDC) were purchased from Sigma (USA). All other reagents were of analytical grade and used as such.

## Methods

### Synthesis and characterization of PLGA-*b*-PEG co-polymer

The PLGA-*b*-PEG co-polymer was synthesized according to reported method[1,2] with slight modification. Amine-functionalized copolymer PLGA-*b*-PEG (PLGA-*b*-PEG-$NH_2$) was synthesized by tethering $NH_2$-PEG-NH-Boc (Mol. wt. 2000 Da) to PLGA-COOH **(Supplementary Fig. S1 online)**. Briefly, PLGA-COOH (Mol. Wt. 20,000; 1 mmol) was dissolved in methylene chloride and converted to PLGA-NHS by reacting with an excess of NHS (4.4 mmol) in the presence of EDC (4.9 mmol). The activated PLGA-NHS was precipitated using an excess of chilled diethyl ether and repeatedly washed with a chilled mixture of diethyl ether and methanol to remove un-reacted residual NHS. The activated PLGA-NHS was vacuum dried and then dissolved (0.05 mmol) in chloroform followed by addition of $NH_2$-PEG-NH-Boc (0.06 mmol). This reaction mixture was stirred for 12 hrs, and co-polymer was precipitated using ice-cold methanol. The co-polymer was washed with methanol to remove un-reacted PEG. The resulting co-polymer was treated with trifluoroacetic acid for 2 hrs to remove Boc group followed by purification via dialysis against de-ionized water for 24 hrs and dried under vacuum. The resulting PLGA-*b*-PEG-

$NH_2$ was terminally functionalized by the free $NH_2$ group. Similarly, PLGA-*b*-mPEG co-polymer was also synthesized using mPEG-$NH_2$ (methoxy-PEG-$NH_2$; Mol. wt. 2000 Da). $^1$H NMR spectroscopy of the PLGA-*b*-PEG-$NH_2$ was carried out at 300 MHz in $CDCl_3$. Gel Permeation Chromatography (GPC) was carried out by a system having a refractive index detector, and the analysis was carried out at room temperature (RT, 27±2$^0$C) using two serially aligned TSKGEL columns. Dimethylformamide (DMF) was used as an isocratic mobile phase with a flow rate of 1 ml/min.

**Preparation and optimization of PLGA-*b*-PEG NPs**

Nanoprecipitation method was utilized for the preparation of amino group functionalized PLGA-*b*-PEG-$NH_2$ NPs[3,4]. Before preparation of drug loaded NPs, optimization of empty PLGA-*b*-PEG-$NH_2$ NPs was carried out by varying different parameters which control the size of NPs e.g. different solvent (acetone and acetonitrile), the various ratio of solvent to water (keeping polymer concentration constant) and different polymer concentration. Acetone and/or acetonitrile solvents were selected due to their miscibility with water and low boiling point so that they can be removed easily during formulation development to avoid toxicity. Solvent to water ratio was varied from 0.1 to 1 i.e. from 1:1 to 1:10 (keeping 10 mg/mL polymer concentration constant). NPs optimization was also carried out by varying polymer concentrations (from 5-20 mg/mL) in the organic phase.

Briefly, the polymer was dissolved in organic solvent (either acetone or acetonitrile). The NPs were prepared by the addition of polymer solution drop wise to de-ionized water (a non-solvent) followed by sonication for 60 sec. The NPs suspension was stirred for 6 hrs at RT to facilitate complete evaporation of the organic solvent (acetone). In the case of acetonitrile, the NPs suspension was stirred for 2 hrs, and the organic solvent was evaporated in a rotary evaporator at reduced pressure. The NPs were purified by centrifugation (15 min at 18,000 rpm). The PLGA-*b*-PEG-$NH_2$ NPs were washed with water thrice and lyophilized. The effects of the different parameters were observed on overall particle size, Pdi and zeta potential of the NPs and were characterized using dynamic light scattering (ZetaSizer Nano ZS90, Malvern Instrument, USA).

**Preparation and optimization of GLM loaded PLGA-*b*-PEG NPs**

The pre-optimized parameters of acetone and acetonitrile formulations from above studies were selected to synthesize GLM loaded PLGA-*b*-PEG-$NH_2$ NPs. The NPs formulations were further optimized on the basis of the drug to polymer ratio for optimum

drug loading. Briefly, GLM was dissolved in organic solvent, either acetone or acetonitrile. The polymer was likewise dissolved in the same solvent and mixed with the drug. The effect of the different drug to polymer ratio (from 1:1 to 1:10) was optimized (keeping polymer concentration constant and varying drug concentration). The NPs were prepared by the addition of drug-polymer solution drop wise to de-ionized water (a non-solvent) followed by sonication for 60 sec. The resulting NPs suspension was processed as discussed above. The drug loaded NPs were characterized for size, Pdi and zeta potential by the same procedure as described earlier. Similarly, GLM loaded PLGA-*b*-mPEG NPs (non-targeted NPs) were prepared using optimized parameters such as type and concentration of solvent as well as drug to polymer ratio. The GLM content in the NPs formulations was determined by the indirect method by analyzing drug concentration in the supernatant (un-entrapped drug) remaining after ultracentrifugation. The GLM concentration was analyzed by HPLC using mobile phase consist of 0.02M phosphate buffer (pH 3.0):acetonitrile (75:25). GLM loaded plain PLGA NPs were also prepared using oil-in-water emulsion solvent evaporation method. Thus, three formulations viz, GLM-PLGA-*b*-PEG-$NH_2$ GLM-PLGA-*b*-mPEG and GLM-PLGA NPs were prepared.

**Ascorbic acid conjugation to PLGA-*b*-PEG-$NH_2$ NPs and its characterization**

Asc ligand was coupled to the surface of GLM loaded PLGA-*b*-PEG-$NH_2$ NPs posing reactive surface amino groups at the terminal end of PEG **(Supplementary Fig. S4 online)**. Firstly, a reactive imidazole carbamate intermediate was synthesized via reacting hydroxyl group of Asc with carbonyldiimidazole (CDI) using the reported method[5,6] with slight modification. For the above reaction, Asc was taken in DMSO, and the solution was stirred at room temperature for 2 hrs under $N_2$ atmosphere to generate a reactive imidazole carbamate. The product was precipitated with chilled diethyl ether, filtered and vacuum dried. The addition of a highly nucleophilic group (e.g. $NH_2$) to the reactive imidazole carbamate removes imidazole to forms a stable carbamate. The PLGA-*b*-PEG NPs possess highly nucleophilic terminal $NH_2$ groups of PEG. The NPs were suspended in 10M borate buffer (pH 11), and reactive imidazole carbamate of Asc was added, and the mixture was stirred at RT for 2 hrs to form carbamate bond between hydroxyl group of Asc and amino group of PEG. The NPs were collected via centrifugation and washed with de-ionized water thrice to remove un-reacted Asc followed by lyophilization. PLGA-*b*-PEG-Asc NPs were characterized for particle size, Pdi and zeta potential using DLS technique. Surface

morphology was evaluated using SEM and TEM. Asc conjugation on PLGA-*b*-PEG NPs was further confirmed by FTIR spectroscopy and thermogravimetric analysis (TGA).

### *In vitro* drug release studies

Intracellular pH of the brain is near to 7.2, and the pH of the blood is approximately 7.4[7,8]. Both the pH matches closely, therefore *in vitro* drug release of various formulations were carried out in PBS (pH 7.4). Drug release from known amounts of GLM loaded plain PLGA, PLGA-*b*-mPEG and PLGA-*b*-PEG-Asc NPs were determined. The dialysis tubes (MWCO 1000 Da, Spectrum Lab Inc., USA) were filled with a 2 mL (equivalent to 5 mg of drug) of GLM loaded different formulations, hermetically tied and suspended in 50 mL of aqueous compartment (PBS pH 7.4) at 37±2ºC with slow and constant magnetic stirring (100 rpm) under sink conditions. At set time points from 0.5 to 168 hr (up to 7 days), aliquots of 1 mL were taken from the medium and instantly replaced with an identical volume of fresh solution. The GLM concentration was determined with the help of HPLC method. The release profile was measured in triplicate. Data acquired from *in vitro* release studies were plotted as the cumulative amount of drug released versus time.

### Cellular internalization studies in SVCT2 expressing cell line

#### *Cell Culture*

The object of the present work was to develop a formulation which can increase drug delivery to the brain by crossing choroid plexus. The idea was to use SVCT2 transporter expressed on choroid plexus to transport the drug to the brain cell. Therefore, the cell line was selected on the basis of expression of plasma membrane-associated SVCT2 transporter for Asc. NIH/3T3 cell line reported to express plasma membrane-associated SVCT2 transporter for Asc[9,10]. Therefore, NIH/3T3 cell line was selected for cellular uptake studies to assess the targeting potential of the developed nanoparticulate formulations.

#### *Cell uptake using fluorescence microscopy*

This assay was carried out to evidence and compare the uptake of non-targeted and targeted NPs formulation by SVCT2 expressing NIH/3T3 cells. The cells were supplemented with 5 mL of culture medium (DMEM; Invitrogen) and maintained in a $CO_2$ incubator at 37±0.5°C and 5% $CO_2$. Rhodamine B (Sigma, USA) was used as fluorescence dye to evaluate cellular uptake. Rhodamine loaded plain PLGA, PLGA-*b*-mPEG, and PLGA-*b*-

PEG-Asc NPs were prepared according to the optimized method for drug loaded NPs. However, rhodamine was used instead of GLM. The NIH/3T3 cells were seeded at a density of 2 x $10^4$ cells/plate in fibronectin coated tissue culture petri dishes containing 1 mL DMEM, incubated and checked under the microscope for 40-50% confluency. The petri dishes were alienated into four groups, each group containing three petri dishes. The cells were incubated for 48 hrs in 5% $CO_2$ at 37°C with changing of the medium following every $12^{th}$ hrs. The medium was replaced then with 2 mL antibiotic-free and serum-free medium. Subsequently, free rhodamine, rhodamine-PLGA NPs, rhodamine-PLGA-*b*-mPEG NPs and rhodamine-PLGA-*b*-PEG-Asc NPs were placed separately in a group of plates. The NPs were incubated with the cells for 0.5 and 1 hr at 37ºC. After incubation, the cells were washed with Hank's Balanced Salt Solution thrice to remove extracellular NPs[11]. The fluorescence attributed to uptake of fluorescently labeled formulation was then qualitatively observed under fluorescent microscope (Olympus, Osaka, Japan).

***Drug uptake using HPLC***

Drug uptake of free GLM and drug loaded formulations (GLM loaded PLGA, PLGA-*b*-mPEG and PLGA-*b*-PEG-Asc) was estimated using NIH/3T3 cells via HPLC. The cells were seeded into fibronectin coated tissue culture petri dishes at a density of 5 x $10^3$ cells/plate containing DMEM medium and incubated at 37°C and 5% $CO_2$ for 48 hr to acquire more than 75% confluency. Free drug and NPs formulations containing 50 µg/mL of GLM were suspended in the medium and added (100 µL) separately into the wells containing NIH/3T3 cells. After the different incubation period, the culture medium was removed. Cells were detached by cell scrapper by adding 2 mL media and collected from the medium in the form of the pellet by centrifugation at 4000 rpm for 15 min. The cells were washed as mentioned above. To the pellets, 500 µL of 0.5% Triton X 100 was added to rupture the cells[12]. Internalized drug into the cells was extracted by addition of dichloromethane followed by vortexing for 15 min, and the mixture was incubated at 25°C for 6 hr. The supernatant was filtered and the drug concentration was detected using HPLC[13,14].

**Biodistribution studies**

The *in vivo* biodistribution studies of GLM from various formulations (free drugs and drug loaded NPs formulations) were carried out via HPLC method. Biodistribution studies were performed for quantitative measurement of GLM in various organs. Animals were divided into five groups, every group containing 9 rats. Group 1 was treated as control. The

details of samples given via tail vein to each group were as follows: Group 1= PBS (pH 7.4); Group 2= GLM solution; Group 3= GLM-PLGA NPs; Group 4= GLM-PLGA-*b*-mPEG; Group 5= GLM-PLGA-*b*-PEG-Asc (1.5 mg/kg equivalent of GLM). Three animals of each group were sacrificed after 1, 6 and 12 hrs post administration. The tissues from brain, lung, liver, kidney, and spleen were carefully removed immediately after sacrificing and weighed. Subsequently, 5 mL of trichloroacetic acid (10% *v/v* in water) was added to one gram each of various tissues and vortexed for 1 min. A small quantity of ethanol was added, homogenized in a tissue homogenizer and centrifuged at 5000 rpm for 20 min. The supernatant was collected and analyzed for GLM content by HPLC.

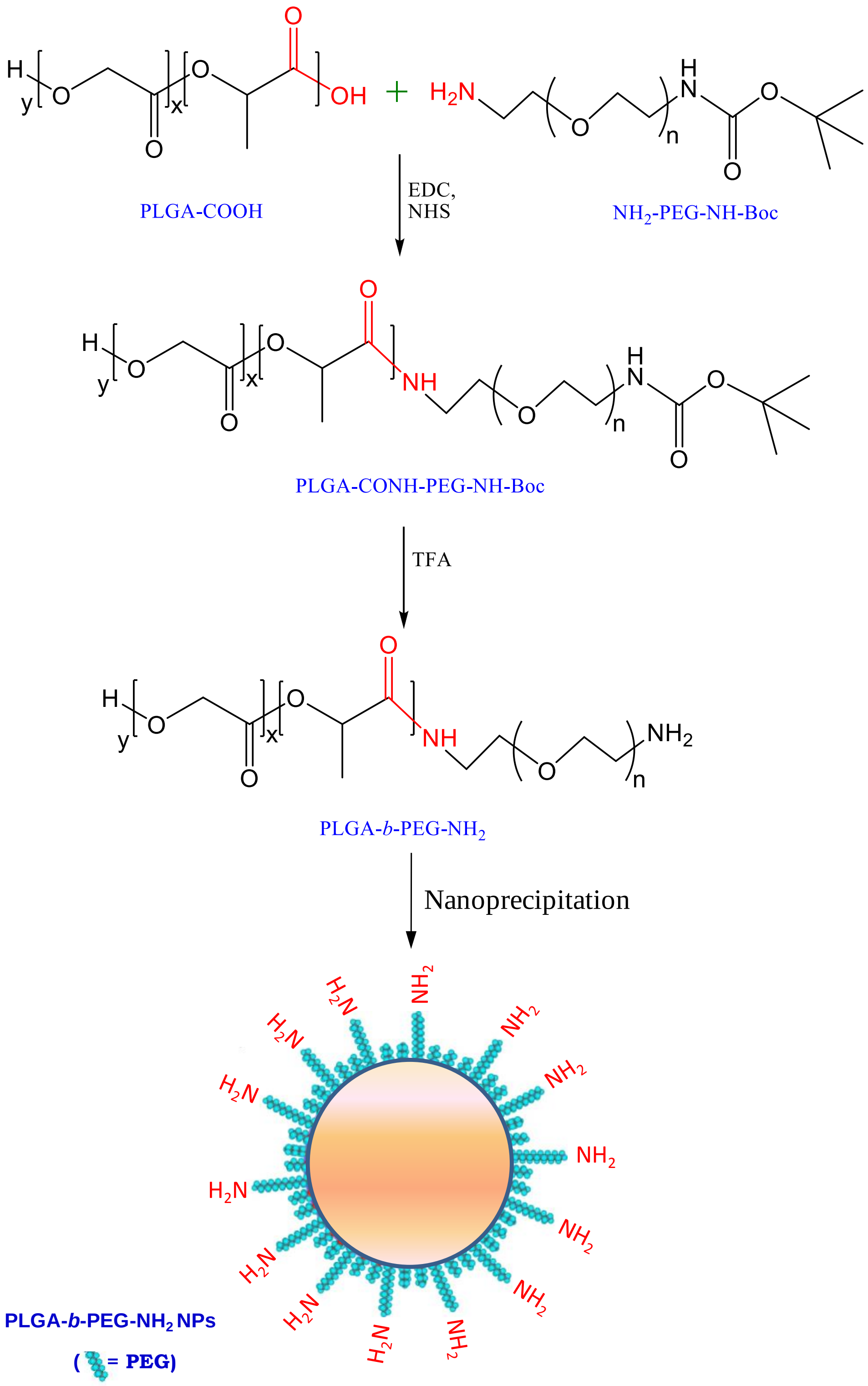


**Supplementary Fig. S1: Synthesis of PLGA-*b*-PEG copolymer and preparation of $NH_2$ terminated NPs.**

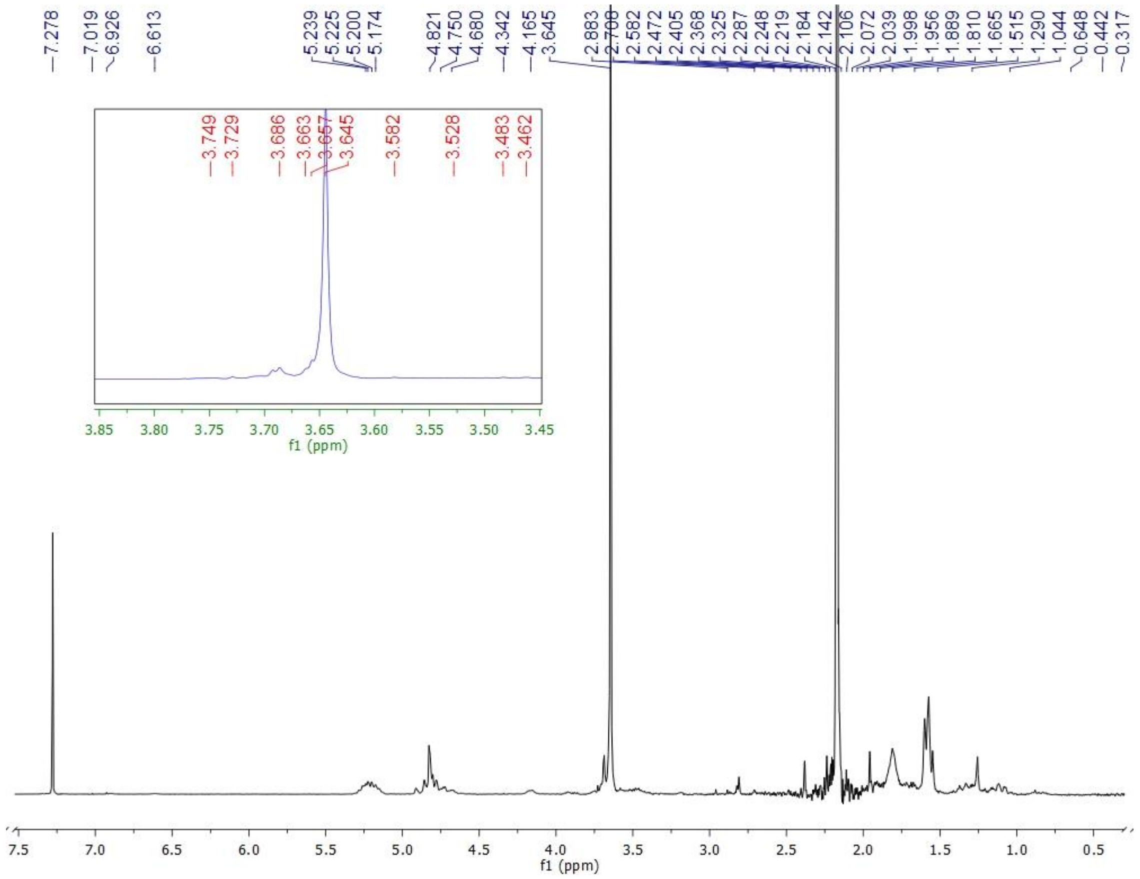


**Supplementary Fig. S2. $^{1}$H NMR spectrum of PLGA-*b*-PEG co-polymer.**

**Supplementary Table S1: Effect of varying type of solvent and solvent:water ratio on PLGA-*b*-PEG NPs.**

| Solvent | Organic to aqueous phase ratio | Particle size (nm) | Pdi | Zeta Potential (mV) |
|---|---|---|---|---|
| Acetone | 01:01 | 112.3±5.7 | 0.118±0.011 | 6.3±0.2 |
| Acetone | 01:02 | 105.7±5.9 | 0.112±0.011 | 5.9±0.1 |
| **Acetone** | **01:05** | **90.1±5.2** | **0.095±0.008** | **4.4±0.1** |
| Acetone | 01:10 | 98.3±4.9 | 0.103±0.008 | 5.4±0.3 |
| Acetonitrile | 01:01 | 137.8±6.3 | 0.147±0.031 | 7.1±0.4 |
| Acetonitrile | 01:02 | 122.5±6.9 | 0.124±0.011 | 6.8±0.5 |
| **Acetonitrile** | **01:05** | **107.7±5.7** | **0.108±0.009** | **5.9±0.2** |
| Acetonitrile | 01:10 | 110.0±7.1 | 0.122±0.016 | 6.1±0.4 |

Values represent as mean±SD (n=6).

**Supplementary Table S2: Effect of varying polymer concentration on PLGA-*b*-PEG NPs.**

| Solvent | Polymer concen-tration (mg/mL) | Particle size (nm) | Pdi | Zeta Potential (mV) |
|---|---|---|---|---|
| Acetone | 5 | 88.3±5.4 | 0.092±0.009 | 4.1±0.2 |
| **Acetone** | **10** | **90.1±5.2** | **0.095±0.008** | **4.4±0.1** |
| Acetone | 15 | 116.7±8.2 | 0.109±0.010 | 6.7±0.3 |
| Acetone | 20 | 133.1±7.5 | 0.132±0.012 | 7.6±0.5 |
| Acetonitrile | 5 | 104.6±4.9 | 0.111±0.009 | 4.5±0.3 |
| **Acetonitrile** | **10** | **107.7±5.7** | **0.108±0.009** | **5.9±0.2** |
| Acetonitrile | 15 | 128.1±6.9 | 0.145±0.011 | 6.8±0.4 |
| Acetonitrile | 20 | 144.8±7.1 | 0.168±0.015 | 7.7±0.3 |

Values represent as mean±SD (n=6).

**Supplementary Table S3: Effect of varying drug to polymer ratio on entrapment efficiency and size of GLM loaded PLGA-*b*-PEG NPs.**

| Solvent | Drug to polymer ratio | % drug entrapment | Particle size (nm) | Pdi | Zeta Potential (mV) |
|---|---|---|---|---|---|
| Acetone | 01:01 | 64.3±4.2 | 93.4±3.6 | 0.108±0.007 | 4.8±0.4 |
| Acetone | 01:02 | 71.8±3.9 | 94.7±5.1 | 0.103±0.009 | 5.0±0.2 |
| Acetone | **01:05** | **80.7±3.5** | **95.5±4.0** | **0.091±0.007** | **5.3±0.2** |
| Acetone | 01:10 | 81.3±5.2 | 109.3±5.1 | 0.112±0.009 | 5.6±0.4 |
| Acetonitrile | 01:01 | 60.5±4.1 | 118.5±6.1 | 0.110±0.011 | 5.7±0.4 |
| Acetonitrile | 01:02 | 66.6±4.7 | 127.0±6.9 | 0.125±0.008 | 6.2±0.3 |
| Acetonitrile | **01:05** | **72.8±3.6** | **135.3±4.2** | **0.149±0.012** | **7.1±0.5** |
| Acetonitrile | 01:10 | 73.7±6.9 | 145.9±6.9 | 0.175±0.014 | 7.5±0.5 |

Values represent as mean±SD (n=6).

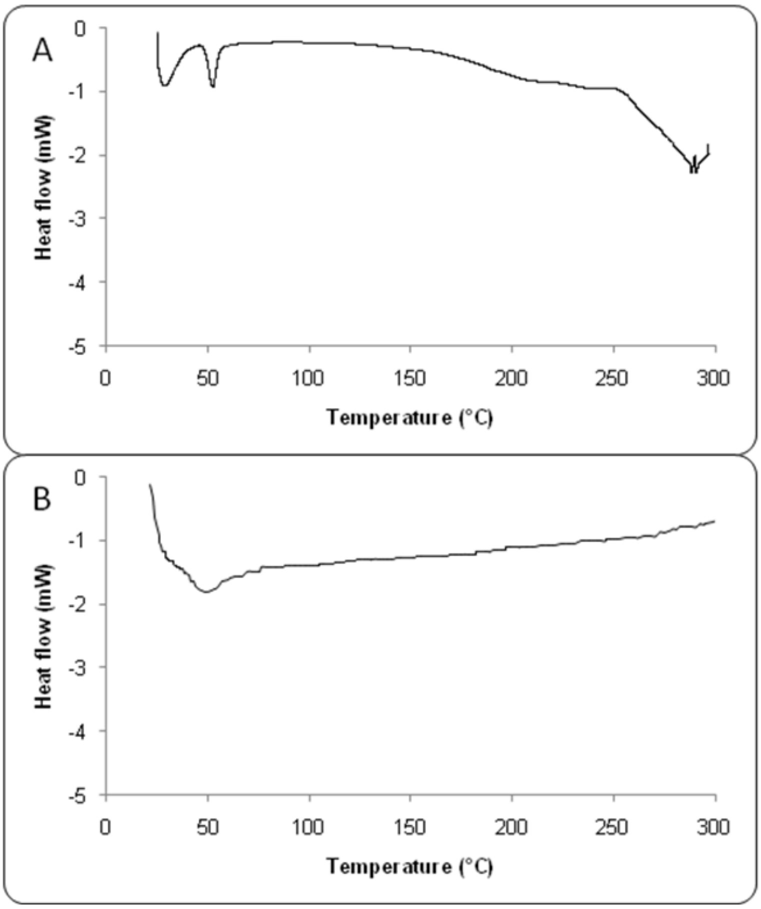


**Supplementary Fig. S3: DSC thermogram of (A) PLGA-*b*-PEG co-polymer and (B) GLM loaded PLGA-*b*-PEG NPs .**

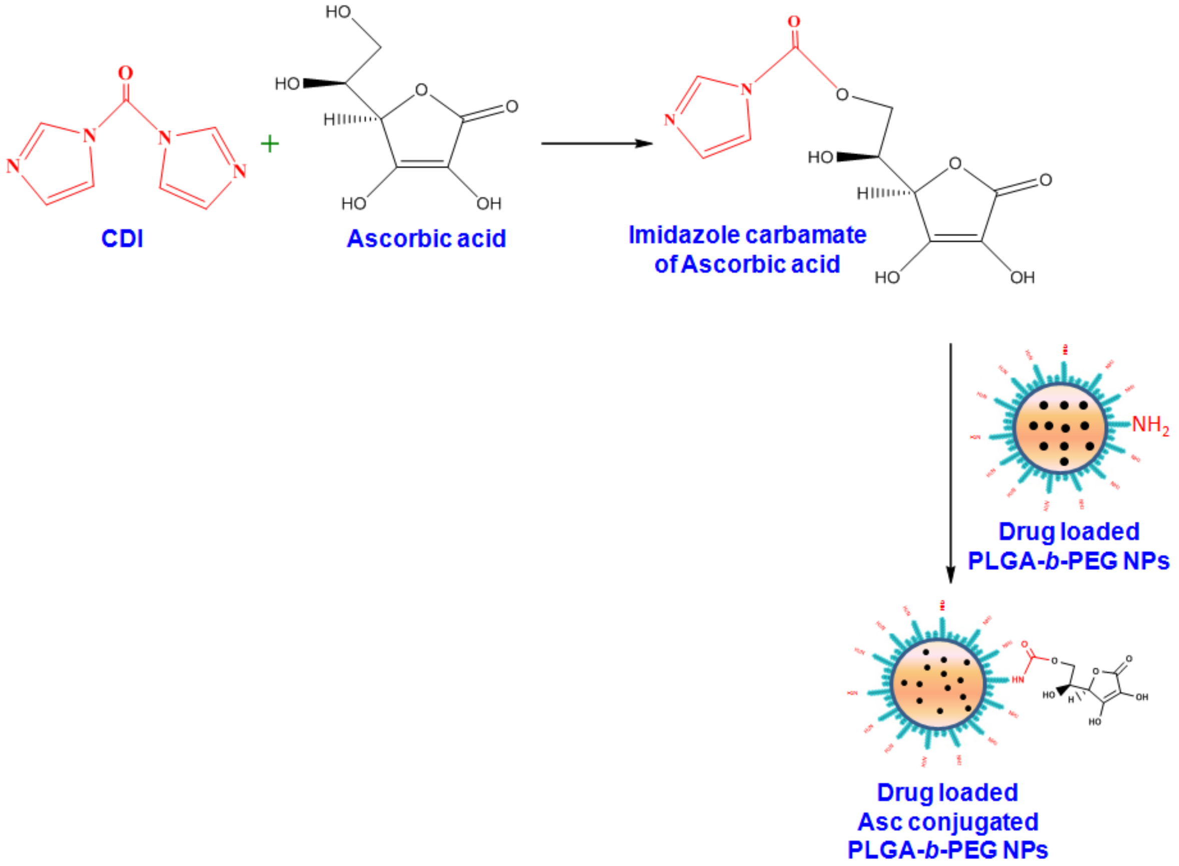


**Supplementary Fig. S4: Preparation of ascorbic acid conjugated to PLGA-*b*-PEG NPs (PLGA-*b*-PEG-Asc NPs).**

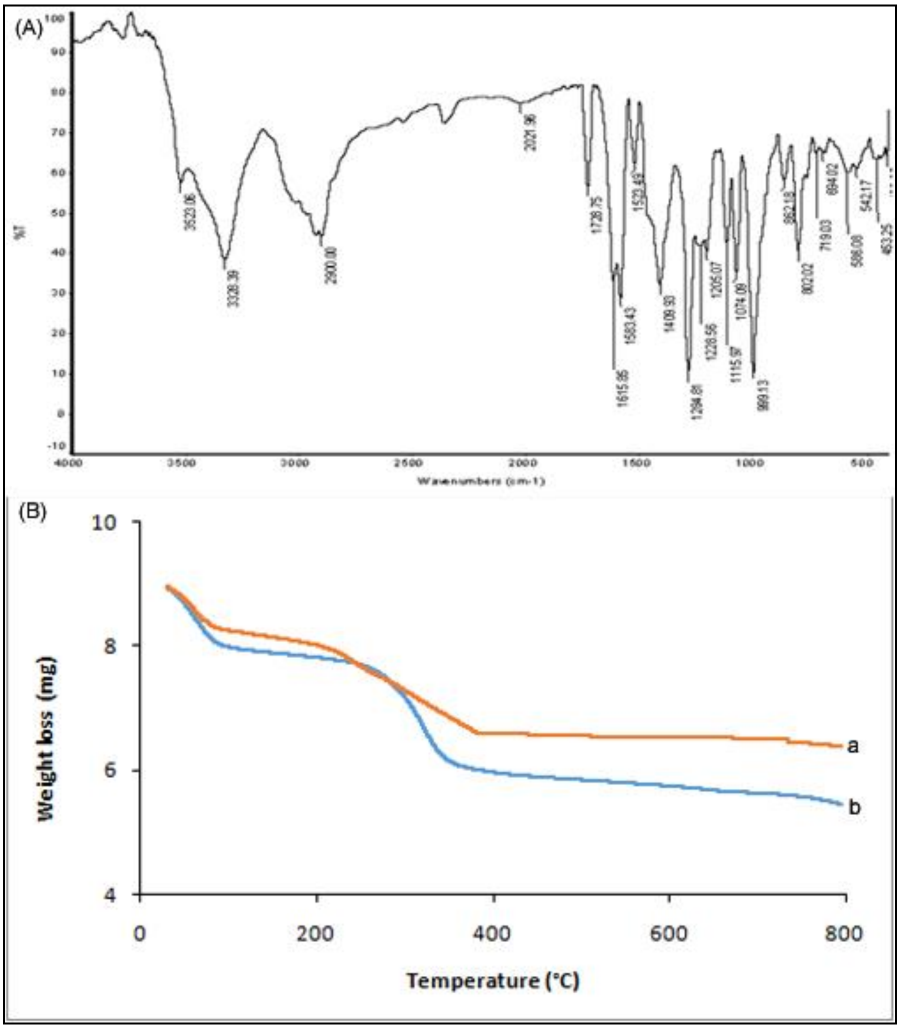


**Supplementary Fig. S5: (A) IR spectrum of PLGA-*b*-PEG-Asc NPs and (B) thermogravimetric analysis of (a) PLGA-*b*-PEG NPs and (b) PLGA-*b*-PEG-Asc NPs.**

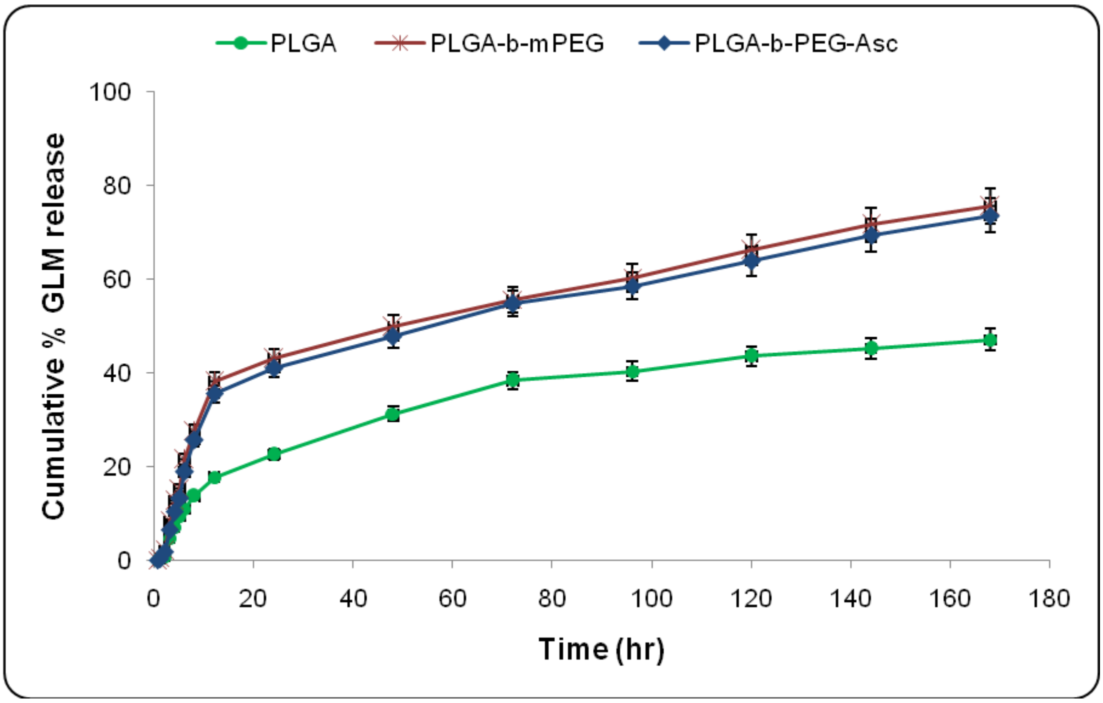


**Supplementary Fig. S6: *In vitro* release profile of GLM from different NPs formulations.**

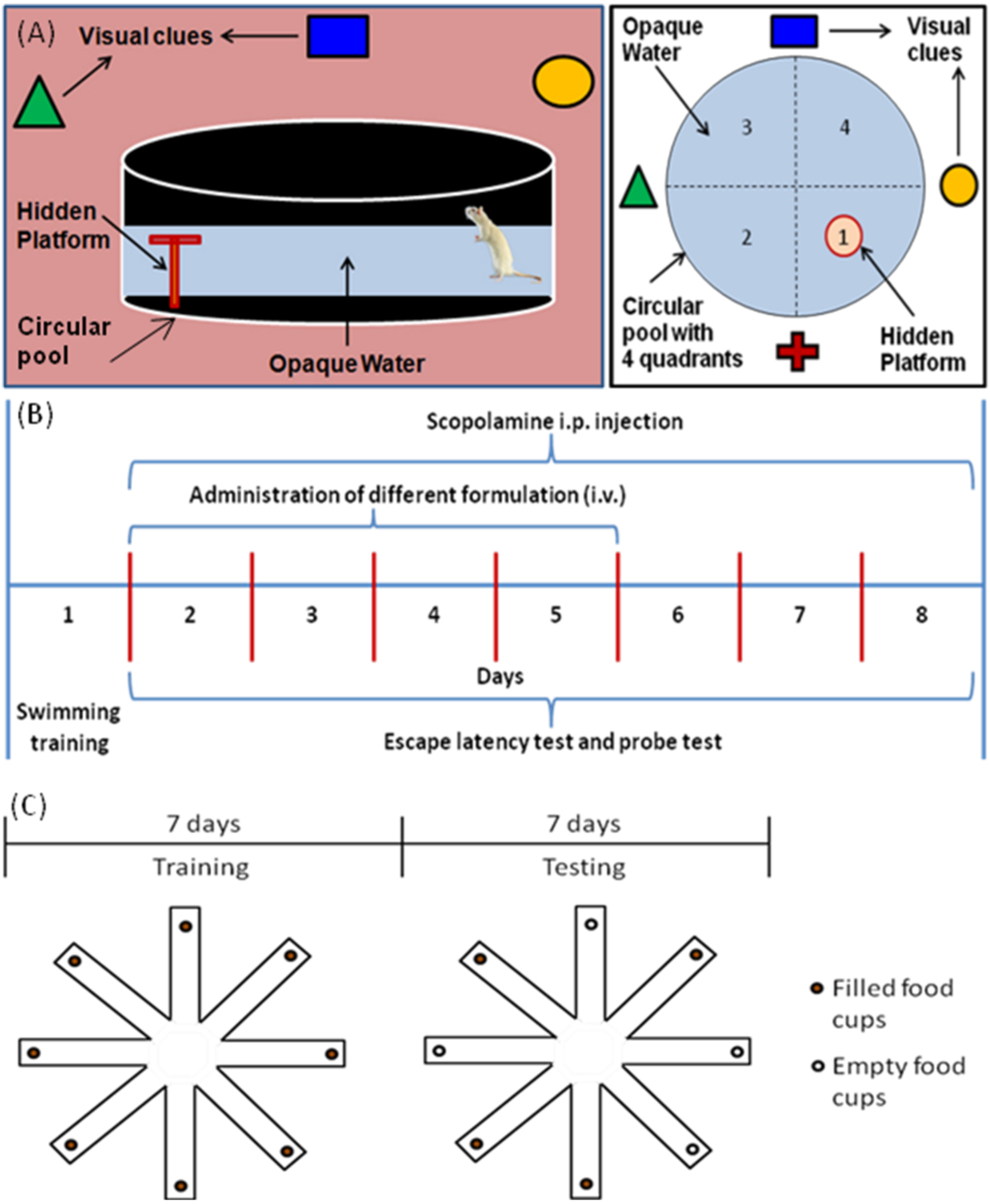


**Supplementary Fig. S7: (A) Schematic drawing of the Morris water maze test, (B) Flow chart of the experimental protocol for the Morris water maze test and (C) Schematic drawing and the flow chart of the experimental protocol for the Radial arm maze test.**

**References**


1. Lin, G., Cosimbescu, L., Karin, N. J. and Tarasevich, B. J. Injectable and thermosensitive PLGA-g-PEG hydrogels containing hydroxyapatite: preparation, characterization and *in vitro* release behaviour. *Biomed Mater.* **7**, 024107 (2012).
2. Cheng, J. *et al*. Formulation of functionalized PLGA–PEG nanoparticles for *in vivo* targeted drug delivery. *Biomaterials* **28**, 869–876 (2007).
3. Danhier, F. *et al*. Targeting of tumor endothelium by RGD-grafted PLGA-nanoparticles loaded with Paclitaxel. *J. Control. Release*. **140**, 166-173 (2009).
4. Tang, L. *et al*. Immunosuppressive activity of size-controlled PEG-PLGA nanoparticles containing encapsulated cyclosporine A. *J. Transplant.* 89614 (2012).
5. Gajbhiye, V. and Jain, N. K. The treatment of Glioblastoma Xenografts by surfactant conjugated dendritic nanoconjugates. *Biomaterials* **32**, 6213-6225 (2011).
6. Patel, S. K., Gajbhiye, V., and Jain, N. K. Synthesis, characterization and brain targeting potential of paclitaxel loaded thiamine-PPI nanoconjugates. *J Drug Target*. **20**, 841-849 (2012).
7. Casey, J. R., Grinstein, S. and Orlowski, J. Sensors and regulators of intracellular pH. *Nat. Rev. Mol. Biol*. **11**, 50-61 (2010).
8. Orlowski, P., Chappell, M., Park, C. S., Grau, V. and Payne, S. Modelling of pH dynamics in brain cells after stroke. *Interface Focus* **1**, 408-416 (2011).
9. Musacchio, V. T. and Salmaso, S. Ascorbate-linked nanosystems for brain delivery. Patent No. CA2756581 A1, Publication date Sep 30, (2010).
10. Hong, S. W. *et al*. SVCT-2 in breast cancer acts as an indicator for L-ascorbate treatment. *Oncogene* **32**, 1508-1517 (2013).
11. Ke, W. *et al*. Gene delivery targeted to the brain using an Angiopep-conjugated polyethyleneglycol-modified polyamidoamine dendrimer. *Biomaterials* **30**, 6976-6985 (2009).
12. Dutta, T. and Jain, N. K. Targeting potential and anti-HIV activity of lamivudine loaded mannosylated poly(propylene imine) dendrimer. *Biochim. Bioph. Act.* **1770**, 681-686 (2007).
13. Karthik, A. *et al*. A study of rivastigmine liposomes for delivery into the brain through intranasal route. *Acta. Pharm.* **58**, 287-297 (2008).
14. Patel, K. B., Patel, A. V., Patel, V. J., Dave, J. B. and Patel, C. N. Quantitative determination of galantamine hydrobromide in pharmaceutical dosage form by RP- high performance liquid chromatography. *J. Chem. Pharm. Res.* **2**, 36-43 (2010).
